# Targeting pyruvate kinase M2 (PKM2) reduces T cell pathogenicity in multiple sclerosis

**DOI:** 10.1016/j.ebiom.2026.106314

**Published:** 2026-05-28

**Authors:** Elena Ellmeier, Alyssa Schnabl, Julia Meissel, Anika Stracke, Sonja Rittchen, Lisa Stöger, Christina A. Passegger, Lena Schwarzl, Cansu Tafrali, Rina Demjaha, Maria Martinez-Serrat, Timothy Kaiser, Lisa-Carina Zeiler, Katharina Seifried, Alina Topler, Isabella Klemen, Bettina Heschl, Anna Damulina, Marah C. Runtsch, Johannes Fessler, Edith Hofer, Michael Khalil, Stefano Angiari

**Affiliations:** a Division of Immunology, Otto Loewi Research Center, Medical University of Graz, Graz, Austria; b Department of Neurology, Medical University of Graz, Graz, Austria; c Division of Oncology, Medical University of Graz, Graz, Austria; d Institute for Medical Informatics, Statistics and Documentation, Medical University of Graz, Graz, Austria; e BioTechMed-Graz, Graz, Austria; f Research Unit Sex and Gender in Disease Pathophysiology, Medical University of Graz, Graz, Austria

**Keywords:** Multiple sclerosis, PKM2, T cells, Immunometabolism

## Abstract

**Background:**

Pyruvate kinase M2 (PKM2) is an enzyme with moonlighting activities that controls murine T cell pro-inflammatory potential in a mouse model of multiple sclerosis (MS). However, no study analysed in detail the expression of PKM2 in human T cell subsets, and whether targeting PKM2 may limit the inflammatory potential of T cells from patients with MS is unknown.

**Methods:**

In this observational, case control study we evaluated the expression of PKM2 in circulating T cells of healthy control individuals (HCs) and patients with MS, as well as its isomerisation in peripheral blood mononuclear cells (PBMCs) from HCs and patients. In parallel, we analysed the impact of targeting PKM2 on the inflammatory profile of T cells from HCs and individuals with MS.

**Findings:**

Circulating effector and memory T cells express higher PKM2 levels compared to naïve T cells, and PKM2 expression in such T cell subsets may correlate with age and disease duration in individuals with MS. Among effector/memory T cells, the Th17/Tc17 subsets display the highest PKM2 expression. Additionally, we observed a preferential inhibition of interferon-gamma (IFN-γ), interleukin-5 (IL-5), IL-13, and IL-17 production by MS T cells upon PKM2 pharmacological targeting. Finally, we found that PBMCs from patients with MS have a higher percentage of PKM2 monomer compared to PBMCs from HCs, supporting heightened PKM2 moonlighting activity.

**Interpretation:**

Our data suggest that PKM2 may represent a therapeutic target to limit T cell-driven inflammation in MS and potentially other human autoimmune diseases.

**Funding:**

Austrian Multiple Sclerosis Research Society, Kulturamt der Stadt Graz, MEFOGraz and Worldwide Cancer Research.

**Research in context**

**Evidence before this study**

Despite research advances in terms of therapies, treating multiple sclerosis (MS) in patients remains challenging. The disease symptoms and progression are heterogeneous between individuals, and new disease biomarkers must be discovered to help patients receive an earlier diagnosis and more specific therapies. New therapeutic targets for the treatment of MS are also needed, especially for some disease forms with limited treatment options, such as progressive MS. Targeting metabolic alterations in immune cells is emerging as a brand-new therapeutic approach to limit their inflammatory and detrimental potential in several contexts, including in autoimmune diseases. This may also hold true for MS, where circulating immune cells display profound metabolic alterations, when compared to healthy individuals. In particular, the enzyme pyruvate kinase M2 (PKM2) represents a valuable target to limit neuroinflammation in animal models of MS, as well as in other models of inflammation and autoimmunity. Targeting PKM2 was shown to limit the pro-inflammatory activity of T lymphocytes, a population of immune cells at the core of the pathogenesis of MS. Nonetheless, how the expression levels of PKM2 are modulated in circulating human T cells is not known. Similarly, whether targeting PKM2 may reduce the detrimental activity of T cells in MS has never been investigated before.

**Added value of this study**

In this study, we evaluated the expression of PKM2 in circulating (blood) human T cells and the effect of PKM2 targeting on T cell function, using three different cohorts of healthy control individuals (HCs) and patients with MS. We report a substantial amount of results suggesting that PKM2 is expressed at high levels in activated T cells, in particular in subpopulations that are highly inflammatory and play a pathogenic role in MS. Additionally, PKM2 expression in specific T cell populations may correlate with disease duration in patients, potentially serving as a biomarker of disease progression. We also show that the pharmacological targeting of PKM2 inhibited the inflammatory activity of T cells more robustly in patients with MS than in HCs, with a stronger inhibitory effect on the production of disease-relevant inflammatory mediators. Finally, we report a differential isomerisation of PKM2 in the PBMCs of individuals with MS, compared to HCs. PBMCs from patients with MS indeed displayed a higher amount of PKM2 monomer, which is usually associated with its non-canonical, pro-inflammatory activity.

**Implications of all the available evidence**

Our study provides evidence for a potential involvement of the enzyme PKM2 in MS. Targeting PKM2 may limit the pathogenic activity of human T cells and may therefore be exploited to limit neuroinflammation and disease progression in patients with MS. Of note, our results may translate to other inflammatory and autoimmune diseases, highlighting the potentially significant clinical value of drugs targeting PKM2.

## Introduction

Multiple sclerosis (MS) is a chronic autoimmune inflammatory disease in which trafficking of blood-borne leukocytes to the central nervous system (CNS) promotes tissue damage, cognitive impairment, and physical disability. MS represents a common cause of physical and cognitive disability in young adults, especially in women, and approximately three million people are affected by the disease worldwide.^1^^,^^2^ The disease typically starts with a single neurological episode caused by inflammation and demyelination (clinically isolated syndrome, CIS). In most of the patients, the disease then moves to a relapsing-remitting phase (RRMS), and after approximately 10–20 years, a progressive clinical course develops in many of the individuals affected, eventually leading to impaired mobility and cognition. However, in some patients, the disease starts from the beginning with a progressive neurodegenerative course.^1^ The clinical presentation is thus heterogeneous, and is related to the spatiotemporal dissemination of lesions and ongoing neurodegeneration within the CNS. Notably, recent studies suggested that MS develops as a continuum of pathophysiological processes that vary between individuals, and highlighted the need for a new diagnostic classification.^3^ Identification of specific biomarkers able to stratify patients according to disease onset, progression, and activity may thus lead to the development of more effective and specific disease-modifying therapies (DMTs). Several DMTs with different mechanisms of action are already available for the treatment of patients with MS. Most of these therapies target the inflammatory response at the core of the disease pathogenesis and effectively manage the more inflammatory forms of MS, such as RRMS.^2^^,^^4^ However, all these drugs come with a spectrum of potential side effects, and there are still only limited therapeutic options for the progressive forms of the disease (PMS),^5^ highlighting the need of identifying novel potential pharmacological targets for disease treatment.

In the last years, studies in the field of immunometabolism revealed that circulating and tissue-infiltrating immune cells involved in the pathogenesis of autoimmunity, in both animal models and human diseases, display an altered metabolic profile that supports their inflammatory potential and pathogenicity.^6^ This holds true also for MS, where the targeting of such metabolic alterations effectively reduced immune cell pro-inflammatory potential in animal models and potentially also in patients with MS.^7^^,^^8^ Among the metabolic mediators suggested to be involved in the pathogenesis of MS, the enzyme pyruvate kinase M2 (PKM2) has recently gained increasing attention. PKM2 is a glycolytic enzyme that can be expressed by cells as a fully functional tetramer or as a monomer/dimer. Studies in tumour cells revealed that the monomeric/dimeric isozyme possesses some moonlighting functions beyond its canonical enzymatic one, including protein kinase activity and translocation into the cell nucleus to regulate gene transcription.^9^ Strikingly, these moonlighting functions were shown to control the functionality of several immune cell subsets, e.g., macrophages, T cells, and dendritic cells (DCs), by promoting the activity of certain transcription factors that usually induce a pro-inflammatory phenotype.^10^, ^11^, ^12^ In particular, we and others have recently shown that murine CD4^+^ T cells upregulate PKM2 expression upon T cell receptor (TCR) activation. Increased PKM2 expression is parallelled by its nuclear translocation, and the pharmacological activation of PKM2 prevented its nuclear import and moonlighting activity in the nucleus, and inhibited the generation of murine T helper 1 (Th1) and Th17 cells. Accordingly, a small molecule allosteric activator of PKM2 limited the development of experimental autoimmune encephalomyelitis (EAE), the mouse model of MS, by reducing the pathogenicity of T cells in the CNS.^11^^,^^13^^,^^14^ These data highlighted the potential of PKM2-based therapies for the treatment of neuroinflammation.

Despite extensive evidence from animal models, the role of PKM2 in human T cell biology and human neuroinflammation is poorly understood. We previously showed that, as in mouse T cells, PKM2 is expressed in human CD4^+^ naïve T cells and, upon TCR activation, it is upregulated and translocates into the nucleus.^11^ We also reported that promoting PKM2 tetramerisation limits the activation of human CD4^+^ T cells *in vitro*.^11^ However, a detailed analysis of PKM2 expression in circulating human T cell subsets is missing. Similarly, whether T-cell PKM2 may play a role in human autoimmune neuroinflammation is unknown. In this work, we analysed the expression of PKM2 in circulating T cells from healthy control donors (HCs) and patients with MS, as well as the overall effect of PKM2 targeting on the inflammatory potential of circulating T cells from HCs (HC T cells) and from patients with MS (MS T cells). We identified associations between the expression of PKM2 in T cells and clinical and demographic parameters in individuals with MS, and we also show that PKM2 is preferentially expressed in effector/memory Th17/T cytotoxic 17 (Tc17) cells and in Th2/Tc2 T cell subsets. This correlates with enhanced inhibition of interferon-gamma (IFN-γ), interleukin-17 (IL-17), IL-5, and IL-13 production by activated T cells of patients with MS upon treatment with an allosteric PKM2 activator, when compared to HCs. Finally, we observed that peripheral blood mononuclear cells (PBMCs) from individuals with MS express higher levels of PKM2 monomer compared to PBMCs from HCs, supporting higher PKM2 moonlighting activity in PBMCs from patients with MS. Our results thus suggest that PKM2 may serve as a biomarker of disease progression in MS, and in particular, that targeting PKM2 may represent a valid approach to reduce T cell inflammatory potential in patients with MS.

## Methods

Detailed information on all reagents and antibodies used in this study are included in Supplementary Table S7.

### Ethics

The study was approved by the ethics committee of the Medical University of Graz (ethical approval numbers: 17-046 ex 05/06, 31-432 ex 18/19, 1131/2024, and 17-291 ex 05/06), and is registered at http://www.clinicaltrials.gov under MarkMS, NCT04892134. All participants signed a written consent form.

### Patient recruitment

24 ml blood were collected from patients with MS who underwent clinical examination at the MS outpatient clinic of the Department of Neurology of the Medical University of Graz. All participants were adults (18 years old or older) and either treatment naïve (‘untreated’) or did not receive any treatment (DMTs or cortisone) for at least six months prior to blood collection (‘previously treated’). Blood was also collected from HCs with no history of neurological diseases and without any immunosuppressive therapy. PBMCs were isolated as previously described^15^ and stored in liquid nitrogen before use. Three study cohorts were used to evaluate PKM2 expression in naïve *vs* memory and effector cells (cohort 1), PKM2 expression in circulating regulatory T cells (Tregs) *vs* effector/memory (E/M) T cell subsets (cohort 2), and PKM2 isomerisation in PBMCs (cohort 3). Characteristics of the cohorts are shown in Table 1, Table 2, Table 3. All recruited individuals were white Caucasian European, and HCs were selected to assure a distribution in sex and age comparable to the MS group. Various parameters were obtained for the timepoint of sample acquisition (baseline), including demographic parameters consisting of age and sex. For patients with MS, we also recorded age at first manifestation, disease duration, disease course, number of relapses, treatment status, and expanded disability status scale (EDSS) score. Additional sociodemographic (e.g., educational level), lifestyle and metabolic (e.g., body mass index [BMI], smoking status, physical activity levels, diet), and environmental parameters (e.g., vitamin D levels) were not routinely collected. Disease courses of MS were defined as CIS, RRMS or PMS.^16^ Relapses were characterised as transient exacerbations of symptoms that last for at least 24 h and occur without any evidence of fever or an infection.^17^ For patients with RRMS, additional parameters including the time between last relapse and baseline and the annualised relapse rate were evaluated. The annualised relapse rate was calculated by dividing the numbers of relapses up to baseline by the disease duration in years.

**Table 1:** Characteristics of study cohort 1.

	Patients with MS	HCs	*p*-value
N	35	26	
females, N (%)	21 (60%)	17 (65.4%)	0.67^a^
age at baseline, mean ± SD	41.74 ± 10.20	39.77 ± 10.08	0.45^b^
CIS, N (%)	9 (25.7%)		
RRMS, N (%)	14 (40.0%)		
PMS, N (%)	12 (34.3%)		
naïve, N (%)	16 (45.7%)		
disease duration in months, median [IQR]	93 [46–196]		
EDSS, median [IQR]; N = 33	2 [0–3.5]		
annualised relapse rate, median [IQR]; N = 14	0.35 [0.22–0.57]		
time from last relapse to baseline in months, mean ± SD; N = 14	52.21 ± 35.24		

MS, multiple sclerosis; HCs, healthy control donors; SD, standard deviation; IQR, interquartile range; CIS, clinical isolated syndrome; RRMS, relapsing-remitting multiple sclerosis; PMS, progressive multiple sclerosis; naïve: untreated patients with MS; EDSS, expanded disability status scale. The annualised relapse rate and the time from last relapse to baseline refer to patients with RRMS (N = 14).

a Chi^2^ test.

b Student's *t*-test. The EDSS of two patients with CIS was not available at time of sampling.

**Table 2:** Characteristics of study cohort 2.

	Patients with MS	HCs	*p*-value
N	25	25	
females, N (%)	17 (68%)	19 (76%)	0.53^a^
age at baseline, mean ± SD	44.40 ± 9.82	43.32 ± 10.94	0.72^b^
CIS, N (%)	5 (20.0%)		
RRMS, N (%)	15 (60.0%)		
PMS, N (%)	5 (20.0%)		
naïve, N (%)	12 (48%)		
disease duration in months, median [IQR]	143 [77–183.5]		
EDSS, median [IQR]; N = 21	1.5 [1–2.5]		
annualised relapse rate, median [IQR]; N = 15	0.26 [0.19–0.40]		
time from last relapse to baseline in months, mean ± SD; N = 14	71.4 ± 63.0		

MS, multiple sclerosis; HCs, healthy control donors; SD, standard deviation; IQR, interquartile range; CIS, clinical isolated syndrome; RRMS, relapsing-remitting multiple sclerosis; PMS, progressive multiple sclerosis; naïve: untreated patients with MS; EDSS, expanded disability status scale. The annualised relapse rate and the time from last relapse to baseline refer to patients with RRMS (N = 15).

The EDSS of three patients with RRMS and one patient with CIS was not available at time of sampling. The time from last relapse to baseline of one patient with RRMS was not available at time of sampling.

a Chi^2^ test.

b Student's *t*-test.

**Table 3:** Characteristics of study cohort 3.

	Patients with RRMS	HCs	*p*-value
N	14	16	
females, N (%)	8 (57.14%)	9 (56.3%)	0.96^a^
age at baseline, mean ± SD	45.71 ± 11.64	41.82 ± 15.39	0.49^b^
naïve, N (%)	2 (14.3%)		
disease duration in months, median [IQR]	206.5 [92–289.3]		
EDSS, median [IQR]; N = 14	2 [1–2.6]		
annualised relapse rate, median [IQR]; N = 14	0.18 [0.12–0.38]		
time from last relapse to baseline in months, mean ± SD; N = 14	115.75 ± 69.35		

RRMS, relapsing-remitting multiple sclerosis; HCs, healthy control donors; SD, standard deviation; IQR, interquartile range; naïve: untreated patients with MS; EDSS, expanded disability status scale.

a Chi^2^ test.

b Mann–Whitney test.

### Analysis of PKM2 expression in T cells by flow cytometry

At the time of the experiments, PBMCs were thawed, washed, counted, and recovered in X-VIVO™ 15 medium (X–VIVO medium; Lonza) for 1 h at 37 °C. 1 × 10^6^ cells were then washed and stained for flow cytometry analysis. Samples were acquired on a BD LSRFortessa and analysed using FlowJo (BD Biosciences). The following protocols, antibody panels and gating strategies were used:

#### Cohort 1

PBMCs were stained with Live/Dead Fixable Red (Thermo Fisher Scientific) according to manufacturer's instructions, and incubated with human serum to block Fc receptors for 10 min on ice. Cells were stained in phosphate-buffered saline (PBS) with the following anti-human antibodies: APC-Cy7 CD3 (BioLegend), PerCP-Cy5.5 CD4 (BioLegend), BUV395 CD8 (BD Biosciences), FITC CD45RA (BioLegend), Pacific Blue CD45RO (BioLegend), and APC CCR7 (BioLegend). Cells were then washed, fixed and permeabilised with the FoxP3/Transcription Factor Staining Buffer Kit (Thermo Fisher Scientific), and stained with an anti-human PE PKM2 antibody (Cell Signalling Technology). Live CD3^+^CD4^+^ and CD3^+^CD8^+^ T cells were gated as follows: naïve (CD45RA^+^CD45RO^−^CCR7^+^), central memory (CD45RA^−^CD45RO^+^CCR7^+^), effector memory (CD45RA^−^CD45RO^+^CCR7^−^), and effector/terminally differentiated (CD45RA^+^CD45RO^−^CCR7^-^) (Supplementary Figure S1). Fluorescence minus one (FMO) staining controls were used to set the gates.

#### Cohort 2

PBMCs were stained with Fixable Viability Dye (FVD) eFluor780 (Thermo Fisher Scientific) according to manufacturer's instructions, and incubated with human serum for 10 min on ice. Cells were then stained in PBS with the following anti-human antibodies: AlexaFluor700 CD3 (BioLegend), PerCP CD4 (BioLegend), BV510 CD8 (BioLegend), PE-Vio770 CXCR3 (Miltenyi Biotec), BV605 CCR4 (BioLegend), BV785 CCR6 (BioLegend), BUV563 CD127 (Thermo Fisher Scientific), and BV711 CD25 (BioLegend). Cells were then washed, fixed and permeabilised with the FoxP3/Transcription Factor Staining Buffer Kit, and stained with the anti-human PE PKM2 antibody. Live CD3^+^CD4^+^ and CD3^+^CD8^+^ effector/memory T cell subsets were gated as follows: Tregs (CD4^+^CD25^+^CD127^neg/low^), Th1/Tc1 (CXCR3^+^CCR4^−^CCR6^−^), Th2/Tc2 (CXCR3^−^CCR4^+^CCR6^−^), and Th17/Tc17 (CXCR3^−^CCR4^+^CCR6^+^) (Supplementary Figure S8). FMO staining controls were used to set the gates.

### Analysis of PKM2 isomerisation and cellular localisation

PKM2 isomerisation and nuclear translocation assays were performed as previously described.^11^ Briefly, PBMCs were collected at day 0 (freshly thawed) or after 3 days of activation in 96-well plates (200,000 cells/well) pre-coated with plate-bound anti-human CD3 and anti-human CD28 antibodies (1.0 μg/ml and 2.0 μg/ml, respectively; BioLegend). In case of anti-CD3/anti-CD28 activation, cells were pre-incubated for 30 min at 37 °C with vehicle (dimethyl sulfoxide; DMSO) or TEPP-46 50 μM (Sigma Aldrich), and then seeded in the plates. DMSO or TEPP-46 were left in the wells for the entire duration of the experiment. For analysis of PKM2 isomerisation, 1 × 10^6^ PBMCs were processed immediately after thawing and recovery. In addition, 3 wells of activated cells were collected at day +3. Cells were washed twice with PBS (pH 8.0), and incubated in PBS (pH 8.0) containing 1 mM disuccinimidyl subaerate (DSS; Thermo Fisher Scientific) for 30 min at 37 °C. The reaction was quenched by adding 1M Tris-HCl (pH 7.5) for 10 min at room temperature (RT), followed by one wash with PBS. For analysis of PKM2 cellular localisation, cells were collected after 3 days (5 wells), and nuclear and cytoplasmic fractions were isolated using the PARIS kit (Thermo Fisher Scientific), according to the manufacturer's instructions.

Crosslinked cells or subcellular fractions were lysed in 4X Laemmli Sample Buffer (Bio-Rad), and boiled for 5 min at 95 °C before loading. Protein samples were resolved by SDS-PAGE and transferred onto a nitrocellulose membrane using the Trans-Blot Turbo RTA Nitrocellulose Transfer Kit (Bio-Rad). Membranes were blocked with 5% (weight/volume) non-fat dry milk (Carl ROTH) in TBST buffer (1X Tris-Buffered Saline, 0.1% Tween-20) for 1 h at RT, and incubated overnight at 4 °C with a primary anti-PKM2 antibody (Cell Signalling Technology), 1:1000 in 5% bovine serum albumin (Carl ROTH) in TBST buffer. After washing, membranes were incubated with an HRP-conjugated secondary antibody (Cell Signalling Technology) at a 1:3000 dilution in 5% non-fat dry milk in TBST buffer and visualised using the Clarity Western ECL Substrate (Bio-Rad) on a ChemiDoc imaging system (Bio-Rad). Protein bands were quantified using Image Lab software (Bio-Rad). β-actin, histone H3 (nuclear loading control), and α-tubulin (cytoplasmic loading control) were used as reference housekeeping proteins (all antibodies from Cell Signalling Technology). For crosslinking analyses, tetramer and monomer band intensities were quantified for each lane, divided to compute tetramer-to-monomer ratio, and summed to obtain total PKM2. The percentage of each oligomer was calculated as its fraction of total PKM2. PKM2 in nuclear fractions was normalised to histone H3, and PKM2 in cytoplasmic fractions was normalised to α-tubulin. Fold change with TEPP-46 treatment was calculated as the normalised treated value divided by the normalised control value for the corresponding fraction.

### Seahorse assay

PBMCs were activated as above with anti-CD3/anti-CD28 antibodies (1.0 μg/ml and 2.0 μg/ml, respectively), in the presence of vehicle (DMSO) or TEPP-46 50 μM (treatment as above). 96-well XF Cell Culture Microplates (Agilent) were pre-coated with poly-d-lysine (50 μg/ml; Thermo Fisher Scientific) one day prior to the assay. Activated cells were collected, washed in the appropriate assay media (see below), counted, and allowed to adhere for 1 h at 37 °C in the absence of CO_2_ prior to the assay. Mitochondrial and glycolytic function were assessed by measuring oxygen consumption rate (OCR) and extracellular acidification rate (ECAR) using a Seahorse XF Pro Analyser (Agilent).

For the Mito Stress Test, Seahorse XF RPMI Medium (Agilent) supplemented with 10 mM glucose (Sigma–Aldrich), 1 mM sodium pyruvate (Sigma–Aldrich), and 2 mM l-glutamine (PAA Laboratories) was used as the assay medium. 100,000 cells/well were analysed using the Seahorse XF Cell Mito Stress Test Kit (Agilent) according to manufacturer's instructions, with injections of oligomycin (1.5 μM), carbonyl cyanide-4-(trifluoromethoxy)phenylhydrazone (FCCP; 1 μM), and rotenone/antimycin A (Rot/AA; both at 0.5 μM) to determine basal respiration, maximum respiration, and spare respiratory capacity following manufacturer's recommendations.

The Glycolysis Stress Test was performed using 50,000 cells/well in assay medium composed of Seahorse XF RPMI Medium supplemented with 2 mM l-glutamine. The ECAR was measured under basal conditions and following sequential injections of glucose (10 mM, Carl ROTH), oligomycin (2 μM; Sigma–Aldrich), and 2-deoxyglucose (2-DG; 50 mM; Sigma–Aldrich) to evaluate glycolysis, glycolytic capacity, and glycolytic reserve following manufacturer's recommendations.

### Analysis of the effect of TEPP-46 on T cell proliferation by flow cytometry

PBMCs were thawed, washed, counted, and recovered in X–VIVO medium for 1 h at 37 °C. Cells were stained with CellTrace Violet (CTV; Thermo Fisher Scientific), following manufacturer's instructions. Cells were then activated with anti-CD3/anti-CD28 antibodies (1.0 μg/ml and 2.0 μg/ml, respectively), in the presence of vehicle (DMSO) or TEPP-46 50 μM (treatment as above). After 5 days, cells were collected, washed, and stained with the following anti-human antibodies: PerCP-Cy5.5 CD4 and BUV395 CD8. Samples were washed twice and acquired/analysed as above. Proliferation and division indices were calculated as previously described.^11^ The gating strategy is shown in Supplementary Figure S6A, while representative proliferation plots from one HC and one individual with MS are shown in Fig. 3A.

**Fig. 1:**
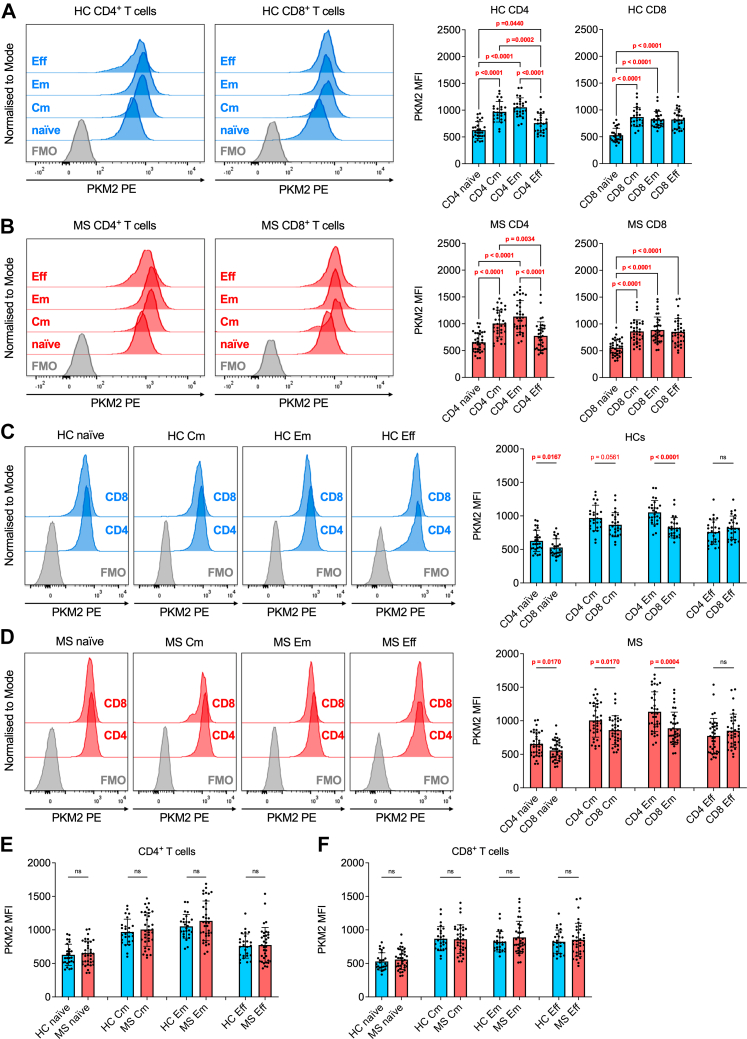
**Effector and memory subsets express the highest PKM2 levels among circulating T cells.** (**A**) Left: representative histogram plots of PKM2 expression in CD4^+^ and CD8^+^ naïve, Cm, Em, and Eff T cells of one HC donor. Right: quantification of PKM2 MFI in CD4^+^ and CD8^+^ T cell subsets of HCs. *p*-values were calculated by one-way Anova test with Tukey's test for multiple comparisons (CD4^+^ T cells) or by Kruskal–Wallis test with Dunn's test for multiple comparisons (CD8^+^ T cells). (**B**) Left: representative histogram plots of PKM2 expression in CD4^+^ and CD8^+^ naïve, Cm, Em, and Eff T cells of one patient with MS. Right: quantification of PKM2 MFI in CD4^+^ and CD8^+^ T cell subsets of individuals with MS. *p*-values were calculated by Kruskal–Wallis test with Dunn's test for multiple comparisons in both graphs. (**C**) Left: representative histogram plots of PKM2 expression in CD4^+^*vs* CD8^+^ naïve, Cm, Em, and Eff T cells of one HC donor. Right: comparison of PKM2 expression between CD4^+^ and CD8^+^ T cell subsets from HCs. *p*-values were calculated by unpaired *t*-test (Cm and Eff cells) or by Mann–Whitney test (naïve and Em cells). (**D**) Left: representative histogram plots of PKM2 expression in CD4^+^*vs* CD8^+^ naïve, Cm, Em, and Eff T cells of one patient with MS. Right: comparison of PKM2 expression between CD4^+^ and CD8^+^ T cell subsets from individuals with MS. *p*-values were calculated by unpaired *t*-test (naïve and Cm cells) or by Mann–Whitney test (Em and Eff cells). (**E**) Comparison of PKM2 MFI in CD4^+^ T cells between HCs and patients with MS. *p*-values were calculated by unpaired *t*-test (naïve, Cm and Em cells) or by Mann–Whitney test (Eff cells). (**F**) Comparison of PKM2 MFI in CD8^+^ T cells between HCs and patients with MS. *p*-values were calculated by unpaired *t*-test (Cm and Eff cells) or by Mann–Whitney test (naïve and Em cells). Data in (**A**–**F**) are the mean ± standard deviation (SD) of 26 HCs and 35 patients with MS. *p*-values <0.05 are displayed in red, bold, while *p*-values <0.1 are displayed in red. ns, non-significant. In (**A**–**B**), ns values are not displayed for visual purposes. In (**A**–**D**): FMO, fluorescence-minus-one staining control.

**Fig. 2:**
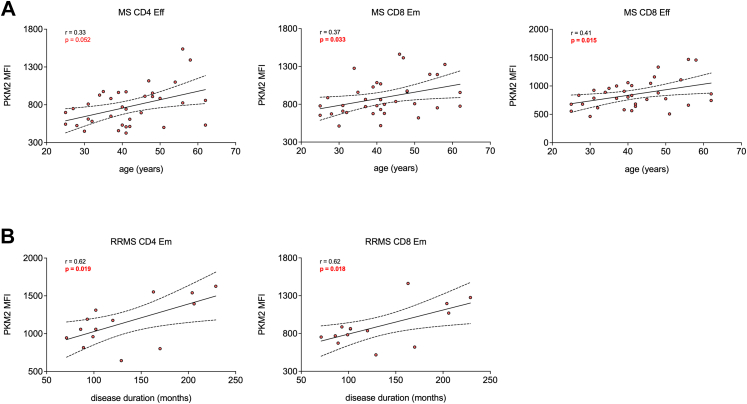
**PKM2 expression levels in Eff and Em T cell subsets positively correlate with age and disease duration in individuals with MS.** (**A**) Correlation analysis between PKM2 expression in CD4^+^ Eff, CD8^+^ Em and CD8^+^ Eff T cells and age of patients with MS at baseline, i.e., at time of blood collection. N = 35. In all graphs, *r* represents the Spearman (MS CD4 Eff and MS CD8 Em) or the Pearson (MS CD8 Eff) correlation coefficient, while *p* represents the *p*-value. (**B**) Correlation analysis between PKM2 expression in CD4^+^ Em and CD8^+^ Em T cells of patients with RRMS and disease duration in months. N = 14. In all graphs, *r* represents the Pearson correlation coefficient, while *p* represents the *p*-value. In (**A**–**B**), *p*-values <0.05 are displayed in red, bold, while *p*-values <0.1 are displayed in red. ns, non-significant.

**Fig. 3:**
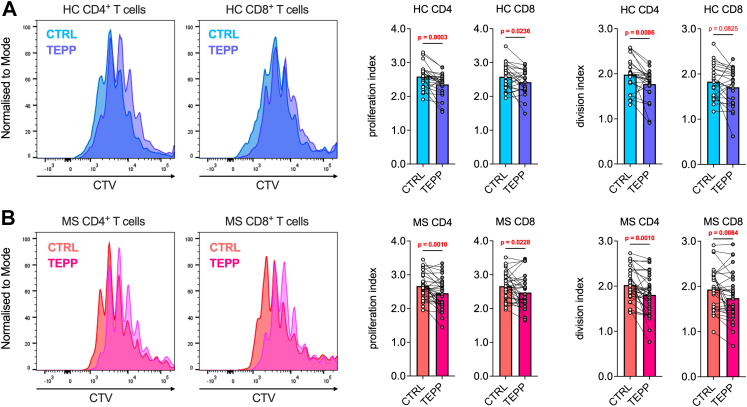
**TEPP-46 inhibits CD4^+^ and CD8^+^ T cell proliferation *in vitro*.** PBMCs from HCs and patients with MS were activated for 5 days with anti-CD3/anti-CD28 antibodies in the presence of vehicle (CTRL) or TEPP-46 50 μM (TEPP). (**A**) Left: representative proliferation plots of CD4^+^ and CD8^+^ T cells of one HC donor. Right: proliferation and division indices calculated from the proliferation assay. *p*-values were calculated by paired *t*-test (proliferation and division index of CD8^+^ T cells) or Wilcoxon test (proliferation and division index of CD4^+^ T cells). (**B**) Left: representative proliferation plots of CD4^+^ and CD8^+^ T cells of one patient with MS. Right: proliferation and division indices calculated from the proliferation assay. *p*-values were calculated by paired *t*-test in all graphs. In (**A**–**B**), data for CD4^+^ T cells are from 25 HCs to 34 individuals with MS. One HC and one patient with RRMS were excluded from the analysis due to technical issues during sample acquisition. Data for CD8^+^ T cells are from 24 HCs to 32 individuals with MS. Indices for one additional HC and two additional patients with PMS could not be calculated due to low cell counts. In all graphs, the bars represent the mean values. *p*-values <0.05 are displayed in red, bold, while *p*-values <0.1 are displayed in red.

### Analysis of the effect of TEPP-46 on cytokine production

PBMCs were thawed, washed, counted, and recovered in X–VIVO medium for 1 h at 37 °C. Cells were activated with anti-CD3/anti-CD28 antibodies (1.0 μg/ml and 2.0 μg/ml, respectively), in the presence of vehicle (DMSO) or TEPP-46 50 μM (treatment as above). After 24 h, cells were collected and pelleted, and supernatants were stored at −80 °C until use. The concentration of the following cytokines was quantified in undiluted supernatants using a customised LEGENDplex™ assay kit (BioLegend): IL-2, IL-4, IL-5, IL-9, IL-10, IL-13, IL-17A, IL-17F, IL-22, IFN-γ, tumour necrosis factor-alpha (TNF-α), granulocyte-macrophage colony-stimulating factor (GM-CSF), and transforming growth factor-beta (TGF-β). The assay was performed according to manufacturer's instructions. In parallel, cells were restimulated with brefeldin A (BFA) 10 μg/ml, ionomycin (iono) 1 μg/ml, and phorbol 12-myristate 13-acetate (PMA) 50 ng/ml (all from Sigma Aldrich) in X–VIVO medium. After 5 h, cells were collected, washed, and incubated with human serum for 10 min on ice. Cells were then fixed in Fixation Buffer (BioLegend), permeabilised with the Intracellular Staining Permeabilization Wash Buffer (BioLegend), and stained in Brilliant Stain Buffer (BD Biosciences) with the following anti-human antibodies: BUV805 CD3 (BD Biosciences), PerCP-Cy5.5 CD4, BUV395 CD8, AlexaFluor488 IFN-γ (BioLegend), PE/Dazzle594 GM-CSF (BioLegend), APC-Cy7 IL-17A (BioLegend), and APC TNF-α (BioLegend). Samples were washed twice and acquired/analysed as above. FMO staining controls were used to set the gates. The gating strategy and representative cytokine plots are shown in Supplementary Figure S6B.

### Analysis of the effect of TEPP-46 on Th cell polarisation and functionality

PBMCs were isolated as above from buffy coats of healthy donors obtained from the Transfusion Medicine Department of the Medical University of Graz. Naïve CD4^+^ T cells were purified by magnetic sorting using the EasySep™ Human Naïve CD4+ T Cell Isolation Kit (STEMCELL Technologies), following manufacturer's instructions. Cells were activated with anti-CD3/anti-CD28 antibodies (1.0 μg/ml and 2.0 μg/ml, respectively; 50,000 cells/well) in X–VIVO medium containing the following cytokines: IL-12 10 ng/ml (R&D Systems) (for Th1 cell polarisation); TGF-β 1 ng/ml (ImmunoTools) + IL-1β 10 ng/ml (PeproTech) + IL-23 100 ng/ml (ImmunoTools) + IL-6 20 ng/ml (ImmunoTools) (for Th17 cells); IL-4 25 ng/ml (ImmunoTools) + anti-human IFN-γ 0.5 μg/ml (BioLegend) (for Th2 cells). Cells were polarised in the presence of vehicle (DMSO) or TEPP-46 50 μM (treatment as above). Th1 and Th17 cells were collected after 5 days, while Th2 cells were collected after 3 days of culture, washed, and re-seeded for 4 additional days in Th2-polarising medium as above + vehicle (DMSO) or TEPP-46 50 μM. Cells and culture supernatants were collected at the end of the polarisation period for flow cytometry, quantitative real-time polymerase chain reaction (qRT-PCR analysis), and enzyme-linked immunosorbent assay (ELISA).

For flow cytometry analysis, cells were restimulated with Cell Activation Cocktail (0.5X; BioLegend) for a total of 4.5 h, with BFA (10 μg/ml; BioLegend) added after 2 h of culture. Cells were then washed and stained with FVD eFluor780, washed again, fixed and permeabilised as above, and stained with the following anti-human antibodies: AlexaFluor488 IFN-γ (Th1 cells); AlexaFluor488 IL-17F and BV650 IL-17A (both from BioLegend) (Th17 cells); APC IL-13 (BioLegend) (Th2 cells). All samples were washed twice and acquired on a CytoFLEX LX Flow Cytometer (Beckman Coulter). Samples were then analysed as above.

For qRT-PCR, cells were pelleted and ribonucleic acid (RNA) was isolated using the PureLink™ RNA Mini Kit (Thermo Fisher Scientific) or the innuPREP RNA Mini Kit 2.0 (IST Innuscreen), according to manufacturer's instructions. RNA was converted to complementary DNA (cDNA) using the High-Capacity cDNA Reverse Transcription Kit (Thermo Fisher Scientific). Gene expression was determined by qRT-PCR using the PowerUp SYBR Green Master Mix (Thermo Fisher Scientific) on the CFX Opus 384 Real-Time PCR System (Bio-Rad). Gene expression was normalised to the reference housekeeping gene TATA-binding protein (Tbp) and calculated using the ΔΔCt method. All primers were purchased from Thermo Fisher Scientific. Primer sequences are reported in Supplementary Table S7.

For ELISA of IL-5, supernatants of Th2 cells were collected after 7 days of culture. IL-5 was quantified using the Human IL-5 DuoSet ELISA (R&D Systems) according to manufacturer's instructions. Absorbance at 450 nm was measured using a SPECTROstar Nano (BMG Labtech). Corrected absorbance values were obtained by subtracting background absorbance, and cytokine concentrations were calculated by extrapolation from a standard curve.

### Analysis of TEPP-46 effect on *in vitro*-induced Treg (iTreg) polarisation and functionality

Human naïve CD4^+^ T cells were activated with anti-CD3/anti-CD28 antibodies (1.0 μg/ml and 2.0 μg/ml, respectively; 50,000 cells/well) in X–VIVO medium + TGF-β 5 ng/ml + IL-2 10 ng/ml (ImmunoTools). Cells were polarised in the presence of vehicle (DMSO) or TEPP-46 50 μM (treatment as above). Cells were collected after 5 days, washed, and stained with anti-human PE CD25 and AlexaFluor647 CD127 antibodies (both from BioLegend). Cells were then washed, fixed and permeabilised with the FoxP3/Transcription Factor Staining Buffer Kit, and stained with an anti-human AlexaFluor488 FoxP3 antibody (BioLegend). To analyse iTreg functionality, total CD3^+^ human T cells were isolated from PBMCs of healthy donors using the Pan T Cell Isolation Kit, human (Miltenyi Biotec) and stained with CTV. iTregs polarised as above were collected at day +5, washed, and mixed with CD3^+^ T cells at different CD3/iTreg ratios (1/0, 1/1, 2/1, 4/1, 8/1 and 16/1). Mixed cells were activated with anti-CD3/anti-CD28 antibodies (1.0 μg/ml and 2.0 μg/ml, respectively; 50,000 total cells/well) and collected after 5 days. Cells were then washed and stained with FVD eFluor780 and with the following anti-human antibodies: PerCP-Cy5.5 CD4, BUV395 CD8, PE CD25 and BV605 CD137 (BioLegend). All samples were washed twice and acquired on a CytoFLEX LX Flow Cytometer. Samples were then analysed as above.

### Statistics

Experimental samples were analysed in a blinded fashion. Data were analysed using Prism 10 (GraphPad) or the R software version 3.6.2 (https://www.r-project.org/). Sample normality was calculated using the Shapiro–Wilk test, and variables were considered normally distributed when the test resulted in a *p* value > 0.05. For normally distributed samples, unpaired or paired Student's *t*-test, or one-way Anova were used to compare two or more groups. For non-normally distributed samples, the non-parametric Wilcoxon, Mann–Whitney or Kruskal–Wallis tests were used. Relevant post-hoc tests for multiple comparisons were applied. Correlation analyses were performed using the Pearson correlation coefficient for normally distributed data with linear relationships or the Spearman correlation coefficient for non-normally distributed data or monotonous relationships. To account for the effects of sex and age, regression or partial Pearson or Spearman correlation analyses were performed. For all parameters, *p* values <0.05 were considered significant. The statistical and post-hoc tests used in each experiment or comparison are specified in the figure legends. Considering its exploratory nature, and that no previous work analysed PKM2 expression and/or functionality in similar study cohorts, a sample size calculation was not performed at the beginning of the study. Analyses were conducted using a complete case approach, and individuals with missing data were excluded from the respective analyses (relevant for cohort 1 and cohort 2—see legends of Table 1, Table 5 and Table 2). Due to the observational nature of our cohorts and the available data, it was not possible to definitely verify whether the missing data were missing at random. Nonetheless, by comparing relevant variables between individuals with complete and incomplete data, we detected no systematic differences in the key variables sex, age, disease duration and annualised relapse rate of the two study cohorts (data not shown), providing support for the missing at random assumption.

### Role of the funding source

Funding sources did not contribute to study design, data acquisition and analysis, and preparation of the manuscript.

## Results

### High levels of PKM2 expression in memory and effector T cells from HCs and patients with MS

We first assessed PKM2 protein expression in T cells from a cohort of 26 HCs and 35 patients with MS (study cohort 1: 9 CIS, 14 RRMS and 12 PMS; Table 1) via flow cytometry. PKM2 expression was evaluated in naïve, central memory (Cm), effector memory (Em), and effector/terminally differentiated (Eff) T cells (Supplementary Figure S1). As expected, virtually all T cells expressed PKM2^11^ (Supplementary Figure S2A). We then quantified the mean fluorescence intensity (MFI) of the PKM2 intracellular staining in each T cell subset, which directly correlates with the levels of protein expression. We first observed that, apart from CD4^+^ Eff T cells, antigen-experienced CD4^+^ and CD8^+^ T cells (Cm, Em, and CD8^+^ Eff) express higher levels of PKM2, compared to naïve T cells, in both HCs and patients with MS (Fig. 1A and B). Surprisingly, we also found that human CD8^+^ T cells express less PKM2 than CD4^+^ T cells, in both HCs and patients, apart from the Eff population (Fig. 1C and D). This differential PKM2 expression was never described before. In individuals with MS, PKM2 levels were comparable in completely naïve (untreated) *vs* previously treated patients (Supplementary Figure S2B). Similarly, in both CD4^+^ and CD8^+^ T cells, PKM2 expression did not differ between individuals with CIS, RRMS and PMS (Supplementary Figure S2C). Interestingly, no significant difference was observed when comparing PKM2 expression between HCs and patients with MS, in all the subsets analysed (Fig. 1E and F), also upon adjustment for sex and age (Supplementary Table S1). These results suggest that PKM2 protein expression in circulating T cell subsets may not serve as a biomarker to distinguish patients with MS from control individuals or patients with different disease forms.

### PKM2 expression in Eff and Em T cells correlates with age and disease duration in patients with MS

We then evaluated a potential correlation between PKM2 expression in T cells and demographics in the study cohort 1. First, we noticed a trend towards higher PKM2 expression in circulating T cells of male donors compared to female donors despite a comparable average age, in both HCs and patients with MS (Supplementary Figure S3A and S3B). Strikingly, when focussing on untreated (naïve) patients, PKM2 levels in T cells from males were significantly higher than in females for most of the subsets analysed (Supplementary Figure S3C). Second, we found that in patients with MS, but not in HCs, PKM2 expression levels positively correlated with age at baseline, i.e., at the time of blood collection (Table 4). The positive correlation was significant for CD8^+^ Em and CD8^+^ Eff T cells, even though a similar trend was observed for most of the subsets analysed (Fig. 2A and Table 4), and was also confirmed upon adjustment for sex (Supplementary Table S2).

**Table 4:** Correlation analysis between PKM2 expression and age in HCs and patients with MS.

Subset	Age at baseline HCs (N = 26)		Age at baseline Patients with MS (N = 35)	
	r	*p*-value	r	*p*-value
CD4 naïve	0.01	0.98	0.27	0.12
CD4 Cm	0.04	0.83	0.18	0.30
CD4 Em	0.06	0.77	0.21	0.24
CD4 Eff	−0.03	0.87	0.33^a^	*0.05*
CD8 naïve	0.20^a^	0.33	0.25	0.15
CD8 Cm	0.08	0.69	0.13	0.47
CD8 Em	−0.07^a^	0.73	0.36^a^	***0.03***
CD8 Eff	−0.10	0.62	0.41	***0.02***

r: Pearson correlation coefficient. *p*-values <0.05 are reported in bold, italic, while *p*-values <0.1 are reported in italic.

a Spearman correlation coefficient.

We then focused specifically on patients with MS, to evaluate whether PKM2 expression may correlate with clinical parameters associated with disease development. When analysing the whole MS cohort, we found no significant correlation between PKM2 expression in T cells and disease duration or the EDSS score (Table 5), also upon correction for sex and age (Supplementary Table S3). However, when focussing specifically on individuals with RRMS, we observed a positive correlation between PKM2 expression and disease duration, which was significant for both CD4^+^ Em and CD8^+^ Em T cells (Fig. 2B and Table 6) and was maintained upon adjustment for sex and age (Supplementary Table S4). In contrast, PKM2 expression did not correlate with either the annualised relapse rate or the time from last relapse to baseline (Table 6 and Supplementary Table S4). Altogether, these results indicate a potential sex- and age-dependent regulation of PKM2 expression in circulating T cells, in particular in patients with MS, and that PKM2 expression in T cells may increase over time in RRMS.

**Table 5:** Correlation analysis between PKM2 expression and disease activity in patients with MS.

Subset	Disease duration (months; N = 35)		EDSS (N = 33)	
	r	*p*-value	r	*p*-value
CD4 naïve	−0.06	0.73	0.11	0.53
CD4 Cm	−0.13	0.47	0.04	0.82
CD4 Em	−0.01	0.96	0.13	0.46
CD4 Eff	−0.18	0.32	0.09	0.64
CD8 naïve	−0.08	0.64	0.11	0.54
CD8 Cm	−0.13	0.45	0.10	0.60
CD8 Em	0.06	0.74	0.18	0.31
CD8 Eff	0.01	0.94	0.23	0.20

EDSS, expanded disability status scale.

r: Spearman correlation coefficient.

The EDSS of two patients with CIS was not available at time of sampling.

**Table 6:** Correlation analysis between PKM2 expression and disease course in patients with RRMS (N = 14).

Subset	Disease duration (months)		Annualised relapse rate		Time from last relapse to baseline (months)	
	r^a^	*p*-value	r^b^	*p*-value	r^a^	*p*-value
CD4 naïve	0.39	0.17	0.20	0.48	0.11	0.71
CD4 Cm	0.52	*0.06*	0.19	0.51	0.01	0.99
CD4 Em	0.62	***0.02***	0.23	0.43	0.16	0.59
CD4 Eff	0.46	0.10	0.22	0.46	0.10	0.72
CD8 naïve	0.50	*0.07*	0.14	0.64	0.09	0.77
CD8 Cm	0.15	0.61	0.10	0.74	−0.17	0.57
CD8 Em	0.62	***0.02***	0.24	0.41	0.37	0.19
CD8 Eff	0.37	0.19	0.27	0.36	0.10	0.75

p-values <0.05 are reported in bold, italic, while p-values <0.1 are reported in italic.

a Pearson correlation coefficient.

b Spearman correlation coefficient.

### An allosteric activator of PKM2 limits its nuclear translocation and human Th17 and Th2 polarisation

We previously demonstrated that PKM2 translocates into the nucleus of human CD4^+^ T cells upon TCR activation.^11^ We also showed that the small molecule TEPP-46, an allosteric activator of PKM2 that induces its tetramerisation and hampers its nuclear translocation, dampens human CD4^+^ T cell activation and proliferation *in vitro*.^11^ However, a detailed analysis of the effect of TEPP-46 on human T cells has never been performed, in particular in T cells from patients with MS. Therefore, in parallel with the analysis of PKM2 expression levels, we also assessed the effect of the molecule TEPP-46 on T cells from HCs and individuals with MS from the study cohort 1 described above. In these experiments, we used TEPP-46 at a concentration of 50 μM, which has been widely used in previous studies.^10^^,^^11^^,^^18^ In preliminary experiments, we also determined that 50 μM was the highest TEPP-46 concentration showing significant inhibition of cytokine production by T cells with no associated toxicity (Supplementary Figure S4A and S4B).

We first confirmed previous observations suggesting that TEPP-46 at a 50 μM concentration induces PKM2 tetramerisation and limits PKM2 translocation into the nucleus of PBMCs activated with anti-CD3/anti-CD28 antibodies (Supplementary Figure S4C and S4D). In parallel, we also measured the effect of TEPP-46 on the real-time metabolic profile of activated PBMCs by using the Seahorse XF Technology. Previous studies on T cells reported contrasting results when evaluating the impact of the knockout or pharmacological activation of PKM2 on the metabolic activity of cultured T cells.^11^^,^^13^^,^^18^ Our results show that, in our system, TEPP-46 had no significant effect on both glycolytic and respiratory activity of PBMCs activated with anti-CD3/anti-CD28 antibodies (Supplementary Figure S4E and S4F). Overall, these results indicate that any effect attributable to TEPP-46 activity is most likely due to its ability to limit PKM2 moonlighting functions.

It was previously shown that TEPP-46 can inhibit the development of mouse Th1 and Th17 cells, as well as the polarisation of iTregs from murine CD4^+^ naïve T cells.^11^^,^^18^ This latter observation may have hindered the translational potential of PKM2 modulation, due to its inhibition of both pro-inflammatory and anti-inflammatory T cells. Therefore, we determined the impact of TEPP-46 on the polarisation of Th1, Th17, and iTreg cells, as well as Th2 cells, from human naïve CD4^+^ T cells. We confirmed that TEPP-46 inhibited human Th17 polarisation, while not impacting human Th1 development (Supplementary Figure S5A and S5C). Interestingly, we also observed that TEPP-46 significantly inhibited the development of human Th2 cells, which has not been previously reported (Supplementary Figure S5B). Importantly, in contrast to the results obtained with murine T cells, TEPP-46 did not inhibit iTreg induction from human naïve CD4^+^ T cells (Supplementary Figure S5D). The functionality of iTregs induced in the presence of TEPP-46 was also comparable to that of iTregs polarised with vehicle (Supplementary Figure S5E). Overall, our data suggest that TEPP-46 may exert an anti-inflammatory effect on type-2 (Th2-mediated) and type-3 (Th17-mediated) inflammation without limiting iTreg generation or functionality. Intriguingly, the latter effect differs between humans and murine models.

### TEPP-46 exerts enhanced inhibitory effect on IFN-γ and IL-17A production by MS T cells

After assessing the overall immunomodulatory and metabolic effects mediated by TEPP-46 on human PBMCs and purified CD4^+^ T cells, we next evaluated its impact on PBMCs from our study cohort 1. First, its effect on T cell proliferation after 5 days of activation was determined. PBMCs were stained with CTV, activated with anti-CD3/anti-CD28 antibodies, and proliferation was quantified by comparing the T cell proliferation and division indices with or without TEPP-46, as previously described.^11^ Our data show that TEPP-46 mildly but significantly decreased both the proliferation and the division index values, compared to vehicle-treated cells (Fig. 3A and B). These results suggest an inhibition of both CD4^+^ and CD8^+^ T cell proliferation by TEPP-46, with a comparable effect between cells from HCs and individuals with MS.

**Fig. 4:**
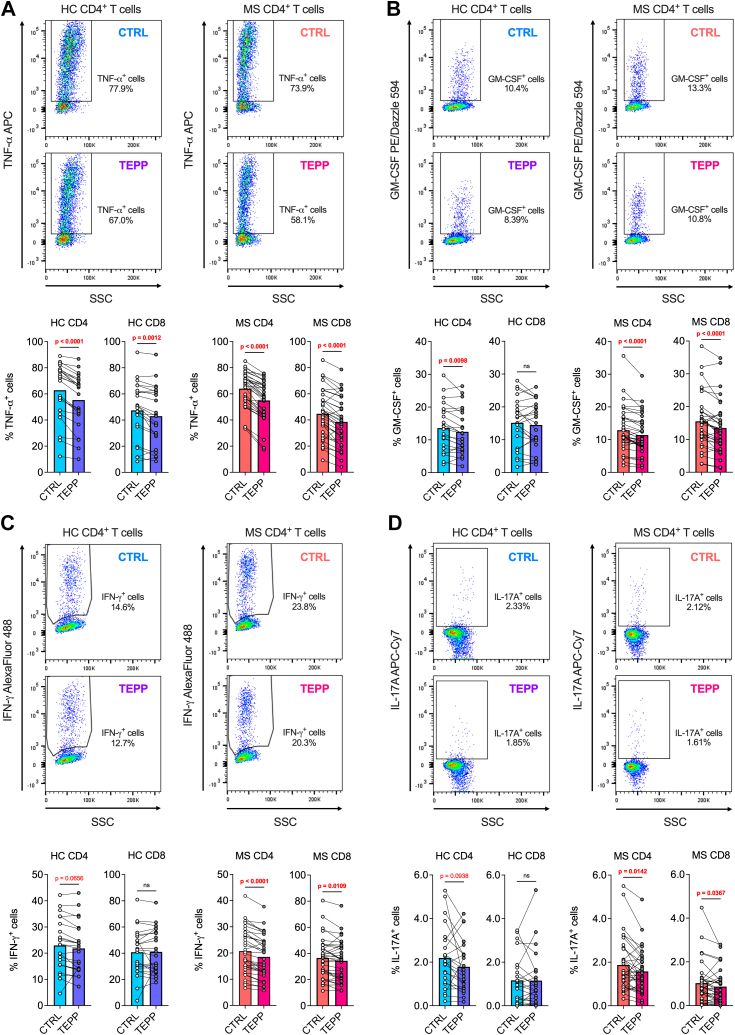
**TEPP-46 treatment leads to a more pronounced reduction of GM-CSF, IFN-γ and IL-17A producing cells in samples from patients with MS.** PBMCs from HCs and patients with MS were activated for 24 h with anti-CD3/anti-CD28 antibodies in the presence of vehicle (CTRL) or TEPP-46 50 μM (TEPP). In (**A**–**D**), top: representative flow cytometry pseudocolour plots of cytokine staining in CD4^+^ T cells of one HC and one individual with MS. Bottom: percentage of cytokine-producing T cells are shown. (**A**) Quantification of TNF-α-producing cells. *p*-values were calculated by paired *t*-test (HC and MS CD8^+^ T cells) or Wilcoxon test (HC and MS CD4^+^ T cells). (**B**) Quantification of GM–CSF–producing T cells. *p*-values were calculated by paired *t*-test (HC CD4^+^ and CD8^+^ T cells) or Wilcoxon test (MS CD4^+^ and CD8^+^ T cells). (**C**) Quantification of IFN-γ-producing T cells. *p*-values were calculated by paired *t*-test in all graphs. (**D**) Quantification of IL-17A-producing T cells. *p*-values were calculated by paired *t*-test (HC CD4^+^ T cells) or Wilcoxon test (all other graphs). In (**A**–**D**), data are from 23 HCs to 35 patients with MS. 3 HCs were excluded from the analysis due to technical issues during sample acquisition. In all graphs, the bars represent the mean values. *p*-values <0.05 are displayed in red, bold, while *p*-values <0.1 are displayed in red. ns, non-significant.

In a second set of experiments, cells were collected after 24 h of activation, and the expression levels of the pro-inflammatory cytokines TNF-α, GM-CSF, IFN-γ, and IL-17A were analysed by flow cytometry. All these cytokines are essential effector molecules in the pathogenesis of MS.^2^ We first found that TEPP-46 reduced the production of TNF-α by both CD4^+^ and CD8^+^ T cells of HCs and patients with MS (Fig. 4A). As TNF-α can be considered as a general cytokine associated with T cell activation, these results, together with the proliferation data, confirm the overall inhibition of T cell activation caused by TEPP-46.^11^ When focussing on more disease-relevant effector cytokines (GM-CSF, IFN-γ, and IL-17A), we noted that their production was significantly inhibited by TEPP-46 in both CD4^+^ and CD8^+^ T cells of individuals with MS. In contrast, TEPP-46 only partially affected cytokine production in CD4^+^ T cells from HCs, while the treatment did not impact cytokine production by CD8^+^ T cells of HCs. This effect was particularly prominent for IFN-γ and IL-17A (Fig. 4B–D). These results suggest that the allosteric activation of PKM2 may inhibit to a greater degree the production of disease-relevant pro-inflammatory cytokines by MS T cells.

### Multiplex analysis reveals a MS-specific effect of TEPP-46 on cytokine production by T cells

To obtain a broader overview of the immunomodulatory effect of TEPP-46, and to potentially confirm its enhanced inhibitory impact on IFN-γ and IL-17A production in MS T cells, the production of 13 cytokines was measured using multiplex assay of supernatants collected 24 h after *in vitro* activation. As expected, there was a trend towards higher production of most cytokines by MS T cells, compared with HCs (Supplementary Table S5). When evaluating the effect of TEPP-46 on cytokine levels, we first found that the production of most cytokines was significantly inhibited by TEPP-46 in samples from both HCs and patients with MS, with minor differences in the percentage of inhibition between the two groups. This was true for IL-2, IL-4, IL-9, IL-10, IL-22, TNF-α and GM-CSF (Supplementary Figure S7), while TGF-β1 concentrations were below the detection level for most patients (data not shown). Overall, these results suggest a broad immunomodulatory effect of TEPP-46 on cytokine production by activated human T cells.

**Fig. 5:**
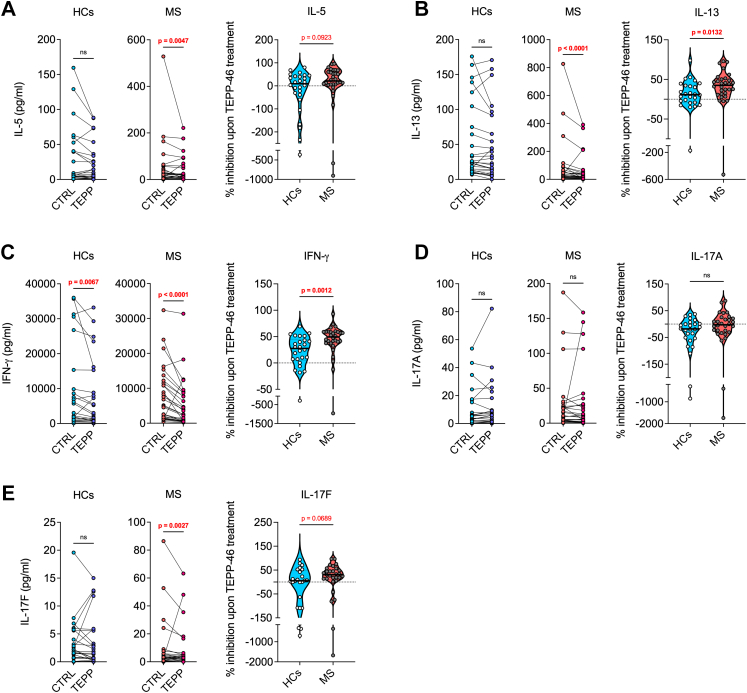
**TEPP-46 inhibits IL-5, IL-13, IFN-γ and IL-17F production more significantly in MS T cells than HCs.** PBMCs from HCs and patients with MS were activated for 24 h with anti-CD3/anti-CD28 antibodies in the presence of vehicle (CTRL) or TEPP-46 50 μM (TEPP). Supernatants were collected and cytokine concentrations were analysed by multiplex assay. In each panel, the left graphs show the cytokine concentrations in supernatants from HC and MS samples, and *p*-values were calculated by Wilcoxon test. The right graph shows the percentage of inhibition upon TEPP-46 treatment, and *p*-values were calculated by Mann–Whitney test. The black horizontal line on top of the violin plots represents the median value. Results for IL-5 (**A**), IL-13 (**B**), IFN-γ (**C**), IL-17A (**D**), and IL-17F (**E**) are shown. Data in (**A**–**E**) are from 26 HCs to 30 patients with MS. Samples from one patient with RRMS, one patient with CIS, and three patients with PMS could not be quantified due to technical issues during sample processing. *p*-values <0.05 are displayed in red, bold, while *p*-values <0.1 are displayed in red. ns, non-significant.

However, TEPP-46 did not significantly inhibit the production of certain cytokines in HC samples, while significant reductions were observed in samples from patients with MS, or cytokine levels were inhibited to a greater extent in samples from individuals with MS than in HCs. First, the production of both IL-5 and IL-13 was exclusively inhibited in samples from patients with MS, with no significant reduction in supernatants from HCs (Fig. 5A and B). Second, while IFN-γ production was significantly lower in samples from both HC and patient cells treated with TEPP-46, compared to control treatment, the reduction was significantly higher in samples from individuals with MS (Fig. 5C), confirming the stronger inhibition of IFN-γ production we observed in MS T cells in the flow cytometry analysis. Surprisingly, while in the flow cytometry experiments we observed a reduction in the percentage of IL-17A^+^ cells upon TEPP-46 treatment, the overall levels of IL-17A were not reduced in the culture supernatants, neither in HCs nor in patient samples (Fig. 5D). Nonetheless, IL-17F was exclusively reduced in samples from individuals with MS (Fig. 5E), supporting a preferential inhibition of type-3 cytokines by TEPP-46 in MS T cells.

### Th17/Tc17 and Th2/Tc2 cells express the highest PKM2 levels among effector/memory T cell subsets

**Fig. 6:**
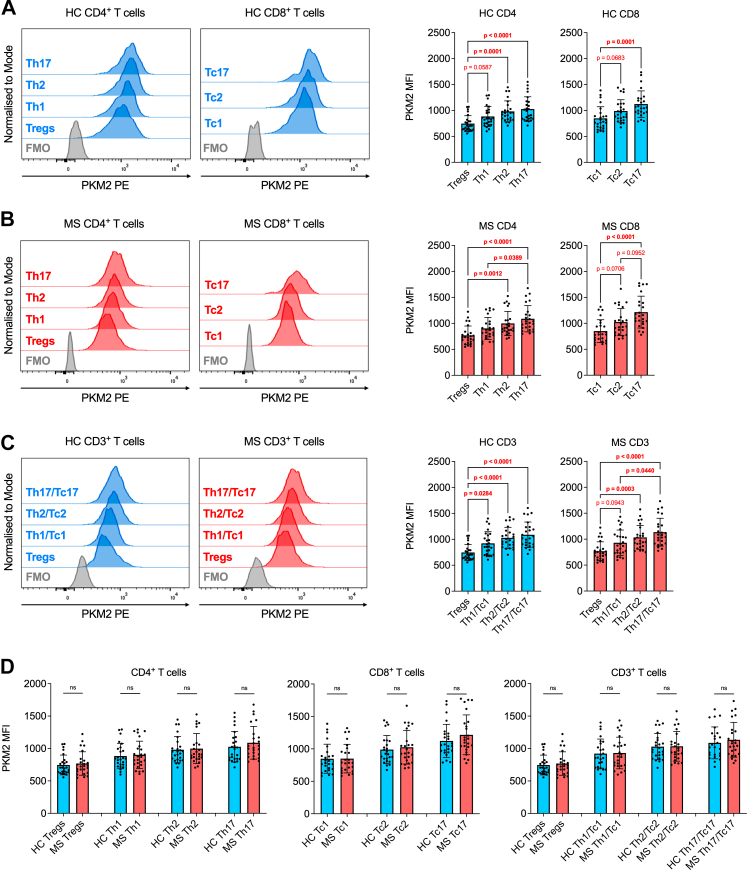
**Circulating Th2/Tc2 and Th17/Tc17 T cell subsets express the highest PKM2 levels.** (**A**) Left: representative histogram plots of PKM2 expression in E/M CD4^+^ and CD8^+^ T cell subsets of one HC donor. Right: quantification of PKM2 MFI in E/M T cell subsets of HCs (**B**) Left: representative histogram plots of PKM2 expression in E/M CD4^+^ and CD8^+^ T cell subsets of one patient with MS. Right: quantification of PKM2 MFI in E/M T cell subsets of individuals with MS. (**C**) Left: representative histogram plots of PKM2 expression in E/M CD3^+^ T cell subsets of one HC and one patient with MS. Right: quantification of PKM2 MFI in CD3^+^ E/M T cell subsets of HCs and individuals with MS. In (**A**–**C**), *p*-values for both CD4^+^ and CD8^+^ subsets were calculated by Kruskal–Wallis test with Dunn's test for multiple comparisons. (**D**) Comparison of PKM2 MFI in CD4^+^, CD8^+^, and CD3^+^ E/M T cell subsets between HCs and patients with MS. All *p*-values were calculated by Mann–Whitney test, apart from the comparison between HC and MS Th1/Tc1 (CD3^+^) cells, which was calculated by unpaired *t*-test. Data in (**A**–**D**) are the mean ± SD of 25 HCs and 25 patients with MS. *p*-values <0.05 are displayed in red, bold, while *p*-values <0.1 are displayed in red. ns, non-significant. In (**A**–**C**), ns values are not displayed for visual purposes. FMO, fluorescence-minus-one staining control.

Considering the stronger effect exerted by TEPP-46 on the production of several effector cytokines by MS T cells, we speculated that this effect may be related to the differential expression and/or activity of PKM2 in different circulating E/M T cells. Therefore, in a second study cohort of 25 HCs and 25 patients with MS (cohort 2: 5 CIS, 15 RRMS and 5 PMS; Table 2) we evaluated the expression of PKM2 in several E/M T cell subsets, based on the presence of specific chemokine receptors and surface markers (Supplementary Figure S8). We first observed that, in both HCs and patients with MS, Th17 (CD4^+^) and Tc17 (CD8^+^) cells expressed the highest PKM2 levels, followed by Th2/Tc2 and Th1/Tc1 cells, whereas Tregs expressed the least PKM2 (Fig. 6A and B). Considering the very low cell number of some E/M populations detected by flow cytometry (e.g., Tc17 cells), we also repeated the same analysis on total CD3^+^ cells (CD4^+^ + CD8^+^ T cells combined), and we obtained similar results, with a trend of higher PKM2 expression in Th17/Tc17 and Th2/Tc2 cells (Fig. 6C). These data may point towards a preferential role of PKM2 in Th17/Tc17 and Th2/Tc2 cell functionality, and suggest that targeting PKM2 *in vivo* may have a more potent impact on such T cell subsets. Surprisingly, we found no differences in PKM2 MFI when comparing the different T cell subsets between HCs and patients (Fig. 6D), also upon adjustment for sex and age (Supplementary Table S6). This result was unexpected, and suggests that the MS-specific effect of TEPP-46 shown in Figs. 4 and 5 may be related to modulation of PKM2 activity, rather than depending on its overall expression. Differently from cohort 1, we did not observe a correlation between PKM2 levels in E/M T cell subsets with either age at baseline or sex (data not shown). When focussing on the MS cohort, we found no differences in PKM2 expression between naïve (untreated) and previously treated patients with MS (Supplementary Figure S9A). Interestingly, patients with CIS and RRMS seem to express higher PKM2 levels than individuals with PMS in all subsets analysed apart from Tregs, although the number of patients with PMS included in the cohort was very low (N = 5) (Supplementary Figure S9B). However, no correlation was observed between PKM2 expression levels and disease duration, annualised relapse rate, EDSS, and time from last relapse, even when only considering patients with CIS and RRMS (data not shown). Overall, results from the analysis of the second study cohort suggest a higher PKM2 protein expression in type-2 (Th2/Tc2) and type-3 (Th17/Tc17) E/M human T cells.

### PBMCs from patients with MS express higher PKM2 monomer at baseline

**Fig. 7:**
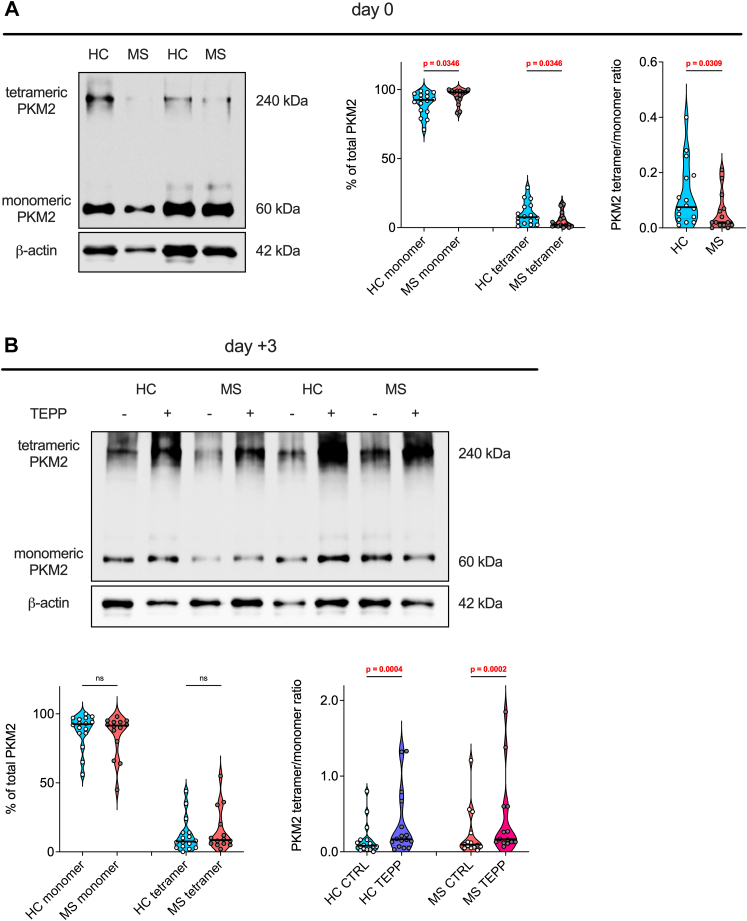
**PBMCs from patients with MS express PKM2 almost exclusively in a monomeric form at baseline.** PBMCs from HCs and individuals with MS were immediately processed upon thawing and recovering (day 0) (**A**) or upon 3 days of *in vitro* activation with anti-CD3/anti-CD28 antibodies (**B**), to induce protein cross-linking. PKM2 expression and isomerisation was then analysed by Western blot. (**A**) Left: representative image showing PKM2 isomerisation (tetrameric and monomeric forms) in PBMCs from 2 HCs to 2 patients with MS at day 0. Right: quantification of the results obtained from 16 HCs to 14 patients. (**B**) Top: representative image showing PKM2 isomerisation in PBMCs from 2 HCs to 2 patients with MS upon 3 days of activation in the presence of vehicle (−) or TEPP-46 50 μM (+). Bottom: quantification of the results obtained from 16 HCs to 14 patients. CTRL: vehicle treatment. TEPP: TEPP-46 treatment. In all graphs in (**A**–**B**), *p*-values were calculated by Mann–Whitney test. ns, non-significant. The black horizontal line on top of the violin plots represents the median value.

Our data on naïve T cell polarisation (Supplementary Figure S5) suggest a preferential effect of TEPP-46 on type-2 and type-3 T cells. Similarly, data in Fig. 6 support a more prominent role for PKM2 in type-2 and type-3 human E/M T cells. Nonetheless, results in Fig. 5 were unexpected, as we did not detect any significant difference in PKM2 expression between the circulating T cells of HCs and patients with MS. However, the role of PKM2 in T cells goes beyond its expression levels, and also depends on its isomerisation, which controls the non-canonical, moonlighting functions.^9^^,^^11^^,^^13^ For this reason, we obtained additional primary samples as a third study cohort, focussing on patients with RRMS (cohort 3: 16 HCs and 14 RRMS, Table 3), to evaluate whether PKM2 isomerisation may differ between PBMCs of HCs and patients with MS. Strikingly, by performing protein cross-linking followed by Western blot analysis,^11^ we found that PBMCs from individuals with MS almost exclusively express PKM2 as a monomer at baseline, while PBMCs from several HCs also have a significant amount of PKM2 tetramer (Fig. 7A). Interestingly, this difference disappeared once PBMCs were activated for 3 days with anti-CD3/anti-CD28 antibodies, and activation in the presence of TEPP-46 induces PKM2 tetramerisation in samples from both HCs and patients (Fig. 7B). Nonetheless, as the PKM2 monomer/dimer is responsible for the moonlighting functions of the enzyme, our data may suggest a more prominent moonlighting activity of PKM2 in circulating leukocytes of patients with MS.

## Discussion

In recent years, several studies highlighted the importance of both enzymatic and non-canonical, moonlighting functions of PKM2 for immune cell activation and inflammatory potential. PKM2 indeed controls the functionality of T cells, natural killer cells, DCs, macrophages, neutrophils, and B cells by regulating signalling pathways, metabolic remodelling and effector functions.^10^, ^11^, ^12^^,^^19^, ^20^, ^21^ However, most of these works focused primarily on murine cells, with limited information on how PKM2 may regulate human leucocyte activity. In particular, whether PKM2 expression differs between circulating human T cell subsets, and whether targeting PKM2 may represent a valuable approach to limit T cell pathogenicity in human diseases, is unknown. We and others recently highlighted the potential of PKM2 as a novel therapeutic target in T cell-mediated autoimmune neuroinflammation.^11^^,^^13^^,^^14^ Nonetheless, the role of PKM2 in human MS has never been investigated.

In this observational, case control and single centre study, we analysed the expression of PKM2 in the main circulating T cell subsets of HC individuals and patients with MS. In a first cohort, we detected higher PKM2 expression in Cm, Em and Eff T cells compared to naïve T cells, in both CD4^+^ and CD8^+^ T cell compartment. The higher PKM2 expression in Em and Eff T cells was not surprising, as PKM2 is upregulated in human T cells upon activation.^11^ However, less expected were the high PKM2 levels in Cm T cells. Cm T cells are generally considered circulating ‘quiescent’ T cells that patrol the body, searching for their cognate antigen. Nonetheless, previous studies have shown that, from a metabolic point of view, Cm T cells have a mixed metabolic activity, showing a ‘pre-primed’ metabolic profile that allows rapid activation upon TCR engagement and underlines their fast and high-effective responsiveness.^22^ Our data suggest that, upon activation and acquisition of a memory phenotype, T cells may maintain high PKM2 expression, which potentially contributes to the rapid metabolic and functional re-activation of Cm T cells upon TCR engagement. Further studies are thus warranted to investigate the importance of PKM2 enzymatic and moonlighting functions for human memory T cell generation and functionality. Our analysis also showed a higher expression of PKM2 in CD4^+^ T cells when compared to their CD8^+^ counterparts, which was never described before. Interestingly, while the impact of PKM2 pharmacological or genetic targeting in CD4^+^ T cells is well known,^11^^,^^13^^,^^14^ how PKM2 regulates CD8^+^ T cell functionality is still unclear. Knockout of PKM2 in CD8^+^ T cells reduced their overall functionality, similar to CD4^+^ T cells, but these studies were mainly performed in murine T cells.^23^^,^^24^ Interestingly, a recent study found that the pharmacological activation of PKM2 boosts the effector functions of murine and human CD8^+^ T cells by promoting mitochondrial functionality.^25^ These results are in contrast with our data showing an inhibitory effect of TEPP-46 on human CD8^+^ T cell proliferation and cytokine production. Of note, in our experiments, the effect of TEPP-46 was generally less pronounced in CD8^+^ T cells than in CD4^+^ T cells, in particular in HCs, which may relate to the lower expression of PKM2 observed in naïve, Cm, and Em CD8^+^ T cells. Further studies are therefore needed to dissect in detail the metabolic and non-metabolic roles of PKM2 in human circulating CD8^+^ T cells.

When correlating PKM2 expression in naïve, Cm, Em and Eff T cells with demographic and clinical parameters in HCs and patients with MS, we observed some interesting trends. First, PKM2 expression seems to be higher in T cells from male compared to female individuals, in particular in untreated patients. Differential PKM2 expression between female and male human immune cells was never reported, and may be of relevance from a functional and therapeutic standpoint.^26^ A wider cohort is needed to confirm this observation. Strikingly, we also found that PKM2 expression in T cells positively correlates with age in individuals with MS but not in HCs, with a similar trend in most of the subsets analysed. Additionally, in patients with RRMS, we observed a positive correlation between disease duration and PKM2 expression, in particular in Em T cells. Interestingly, we did not observe such correlations when analysing PKM2 levels in specific E/M T cell subsets (cohort 2). This partial discrepancy may be explained by the fact that memory cells gated in cohort 1 may also contain other T cell populations with an E/M phenotype, such as senescent T cells or CD20^+^ T cells.^27^^,^^28^ Of note, recent studies started to implicate both these T cell subsets in the pathogenesis of MS.^29^^,^^30^ Evaluation of PKM2 expression in such T cell populations may therefore be of relevance. Overall, a higher number of patients must be analysed to confirm these results, which nonetheless may suggest that, in RRMS, systemic circulating factors may induce and sustain PKM2 upregulation in T cells. How PKM2 expression is regulated in T cells by soluble mediators such as pro-inflammatory cytokines is not known, but previous work showed that it may be upregulated in other cell types upon treatment with cytokines relevant for the pathogenesis of MS, such as IL-17A.^31^ Importantly, results of the correlation analyses were confirmed upon adjustment for sex and age, the two demographics parameters we collected from the enrolled patients. As highlighted in the Methods section, we did not collect additional sociodemographic, lifestyle, metabolic and environmental parameters. Variables such as diet, BMI, smoking status, and vitamin D levels, among others, have been reported as confounding factors when analysing and correlating some experimental results in MS research,^32^^,^^33^ and should be considered in follow-up studies evaluating the role of PKM2 in MS.

Our study also shows a heightened impact of a PKM2-targeting drug on the inflammatory potential of MS T cells when compared to HC T cells, highlighting the promising translational value of such a pharmacological approach in MS. By using a small molecule allosteric activator of PKM2 named TEPP-46, which induces PKM2 tetramerisation and limits its nuclear translocation^11^^,^^34^ (Supplementary Figure S4), we found that, as expected, this drug has a global immunomodulatory effect on both HC and MS T cells, blunting their proliferation and the production of many effector cytokines. However, the most important finding was the stronger inhibition of IFN-γ, IL-17A/F, IL-5, and IL-13 production by MS T cells, when compared to HC T cells, upon TEPP-46 treatment. Inhibition of IFN-γ and IL-17 production by circulating MS T cells represents a promising result when considering the translational value of molecules targeting PKM2 in patients. Such cytokines are indeed well-known effector players in MS pathogenesis.^2^ On the other hand, the exclusive inhibition of IL-5 and IL-13 production in MS T cells *vs* HC T cells, despite being striking, is of difficult interpretation. IL-5 and IL-13 are essential effector cytokines in type-2 responses, such as allergy and asthma.^35^ Previous studies have attempted to elucidate their role in MS, but results are contrasting, and a consensus in the field has not yet been reached. IL-5 is generally considered an anti-inflammatory cytokine, and some works correlated circulating IL-5 levels to lower inflammation and treatment response in patients with MS.^36^^,^^37^ However, a recent work suggested that IL-5 may favour B cell development in patients, cells that are at the core of disease pathogenesis.^38^ Similarly, IL-13 was also initially thought to play a protective role in MS, but more recent studies identified CNS-infiltrating IL-13^+^ T cells as potential pathogenic players in patients with MS (discussed in ref. 39). Of note, also Th2 cells were thought to represent a protective population in MS, but this concept has been recently challenged.^40^ How Th2 cells, and in particular IL-5 and IL-13, regulate neuroinflammation in MS is thus still unclear. Whether the inhibition of IL-5 and IL-13 production upon PKM2 targeting may be beneficial or detrimental in MS must therefore be clarified.

Based on the results obtained from these functional experiments, we analysed the expression of PKM2 in specific circulating E/M T cell subsets in a second study cohort. Our results indicated that, among E/M T cell populations, the type-2 (Th2/Tc2) and type-3 (Th17/Tc17) subsets express the highest PKM2 levels, whereas circulating Treg cells display the lowest PKM2 expression. These data suggest a potential preferential effect of PKM2 targeting *in vivo* on type-2 and type-3 T cell populations. Interestingly, we observed an almost exclusive inhibition of type-2 (IL-5 and IL-13) and type-3 (IL-17) cytokines in patients with MS, compared to HCs, suggesting that this effect may be more pronounced in individuals with MS. The stronger inhibition of IFN-γ production by cells from patients with MS may also be related to an effect on type-3, CXCR6^+^ Th17-like E/M T cells, which can also produce such a cytokine.^41^ Nonetheless, the more potent effect of TEPP-46 on MS T cells was somehow unexpected, as PKM2 levels did not differ between HC and MS T cells in any of the subsets analysed, in both cohort 1 and 2. However, in a third, final study cohort, we identified a striking difference in the isomerisation of PKM2, with the enzyme being present almost exclusively as a monomer in PBMCs of individuals with MS, while PBMCs of most HCs also express a significant amount of PKM2 tetramer. These results point towards a potentially greater moonlighting activity of PKM2 in leukocytes from patients with MS, and may suggest that, in such cells, PKM2 may also preferentially localise in the nucleus or translocate into it at higher levels upon TCR activation, regulating specific signalling pathways important for the production of IL-5, IL-13, IL-17, and IFN-γ. As an example, PKM2 has been shown to directly or indirectly control the phosphorylation of signal transducer and activator of transcription 3 (Stat3), Stat4, and Stat5 in T cells,^11^^,^^13^^,^^42^ and a previous study reported higher STAT protein phosphorylation in MS T cells *vs* HC T cells upon *ex vivo* activation with IFN-γ or IFN-α.^43^ PKM2 targeting with TEPP-46 may preferentially or more strongly inhibit the phosphorylation of specific STAT proteins in MS T cells, which in turn would blunt the expression of specific effector cytokines. Unfortunately, we had access to a limited amount of PBMCs from each donor, and these analyses were not feasible for this study. Such mechanistic experiments would indeed also require evaluation of post-translational modifications, isomerisation, and cellular localisation of PKM2 in PBMCs and purified T cells of patients with MS and HCs. These analyses will be performed in follow-up works.

A preferential role for PKM2 moonlighting activity in type-2 and type-3 T cells was also confirmed in our experiments with naïve CD4^+^ T cells, where TEPP-46 selectively inhibited Th17 and Th2 polarisation, without affecting Th1 cells. Importantly, while reducing T cell inflammatory profile, TEPP-46 inhibited neither the development nor the functionality of iTregs from naïve CD4^+^ T cells. This observation is crucial when considering the potential use of PKM2 allosteric activators in humans, yet surprising. We and others have indeed shown that TEPP-46 blocks iTreg induction from murine naïve T cells *in vitro*, by limiting TGF-β signalling and phosphorylation of Stat5.^11^^,^^18^ The reason for this discrepancy is unclear, even though previous studies highlighted the metabolic differences between mouse and human Treg cells.^44^ Our data confirm that immunological processes may differ between human and murine cells and the importance of a translational approach.

Considering the limited availability of PBMCs, in particular from patients with MS, in our work we used a single concentration of TEPP-46 (50 μM) that we identified in preliminary experiments as the highest non-toxic and effective one. Similarly, specific timepoints for analysis of cytokine production, T cell proliferation, and Th cell polarisation were selected based on preliminary and previous work, to optimise the experimental approach based on the available material. This may appear as a caveat limiting the translational value of our study, and requiring additional analysis of time- and dose-dependent effects of TEPP-46. However, it is important to highlight that TEPP-46 has not been developed for clinical use, and at the concentration we used, its efficacy may be limited. In our hands, inhibition of T cell proliferation was indeed significant but mild (Fig. 3). Nonetheless, cytokine production was robustly dampened by TEPP-46, especially in the supernatants from cultures of MS PBMCs, in which the concentration of most cytokines was reduced by approximately 50% when compared to vehicle treatment (Fig. 5 and Supplementary Figure S7). TEPP-46 thus represents a valuable tool to evaluate the immunomodulatory impact of PKM2 tetramerisation on T cell functionality *in vitro*, yet lacks clinical translational value. Of note, the company Sitryx has recently announced the start of a phase I clinical trial to test the safety of a PKM2 allosteric activator for the treatment of atopic dermatitis.^45^ This confirms that PKM2 is emerging as a promising target for the treatment of T cell-mediated inflammation, and that selective and highly effective allosteric PKM2 activators may represent innovative therapeutic tools in inflammation and autoimmunity. Importantly, as described above, these drugs may affect the functionality of many different immune cell subsets, including neutrophils, DCs, B cells, monocytes/macrophages and T cells. This may cause not only a beneficial reduction in systemic inflammation, but potentially also immune suppression in the treated individuals. Clinical studies such as the one mentioned above are therefore crucial to evaluate the safety, dosage, biodistribution, half-life, treatment regimens and overall immunomodulatory effects of PKM2-targeting therapies in patients.

Our work thus supports PKM2 targeting as an innovative strategy to inhibit T cell pathogenicity in MS. Further studies are needed to determine the mechanistic basis of such an inhibition. It would also be important to confirm whether PKM2 expression in T cells may indeed serve as a biomarker of disease activity and progression in MS, correlating with disease onset, progression, and prognosis in patients. Parameters such as evidence or no evidence of disease activity (NEDA)^46^ and progression independent of relapse activity (PIRA)^47^ should be evaluated, to correlate PKM2 expression in different T cell subsets with new CNS lesions quantified via magnetic resonance imaging. The potential of targeting PKM2 as therapeutic approach may also be of interest for other autoimmune diseases. A recent work in a small cohort of patients with active rheumatoid arthritis (RA) showed that PKM2 mRNA levels were higher in circulating CD4^+^ T cells from individuals with RA, compared to HCs, and that PKM2 levels in T cells positively correlated with serum IL-17A concentrations and with the disease activity score.^48^ This work thus confirms that T-cell PKM2 may represent a valuable therapeutic target also in other human autoimmune diseases.

In conclusion, our results are a first step in the evaluation of T-cell PKM2 as a potential biomarker and/or therapeutic target in MS, and support the concept that targeting PKM2 in T cells may impact human neuroinflammation. Nonetheless, we are aware of the limitations of our work. First, this is a single centre study, and confirmation of our observations in larger and more diverse patient cohorts from different centres is needed. Second, we used PBMCs rather than isolated T cells to analyse the effect of TEPP-46 in HCs and patients with MS. In this setting, we cannot exclude that TEPP-46 may alter the activity of other immune cells (B cells, monocytes, etc.), which would then indirectly impact T cells. Analysis of isolated T cells from HCs and patients with MS should therefore be implemented to confirm our results. Of note, while we only used blood PBMCs in our study, evaluation of PKM2 expression and targeting in T cells isolated from the cerebrospinal fluid of individuals with MS would provide a stronger background for the use of PKM2 allosteric activators as DMTs in MS. Finally, a longitudinal analysis of PKM2 levels and activity in T cells upon patient treatment with DMTs should also be performed. All these follow-up studies are necessary to support the use of PKM2 as a potential biomarker and/or therapeutic target in human autoimmune neuroinflammation.

## Contributors

EE, ASchnabl, JM, AStracke, SR, LStöger, CAP, LSchwarzl, CT, RD, MM-S, TK, L-CZ, KS, AT, IK, MCR, EH, MK, and SA contributed to investigation and formal analysis. BH, AD, EH, MK, and SA contributed to resources. MK and SA contributed to conceptualisation, supervision, and project administration. SR, MK, and SA contributed to acquisition of funding. EE, ASchnabl, SR, MCR, JF, EH, MK, and SA contributed to manuscript writing–original draft. EE, ASchnabl, JM, MCR, EH, MK, and SA contributed to manuscript writing–revised manuscript. EE, MK, and SA accessed and verified the underlying data. All authors read and approved the final version of the manuscript.

## Data sharing statement

Anonymised data sets generated and/or analysed during the study are available from the corresponding authors upon reasonable request. Data sharing will be subject to approval by the relevant committees of the Medical University of Graz and will require signing a data sharing agreement to access the requested data.

## Declaration of interests

All authors have no conflicts of interest to disclose. SR, JF, and MK received additional support from the Austrian Multiple Sclerosis Research Society. AD and RD received support from Novartis, Sanofi, and Janssen (Johnson & Johnson) for conference attendance. RD received additional support from the European Committee for Treatment and Research in Multiple Sclerosis (ECTRIMS) and the BraYn Association for conference attendance (travel grants). SR was awarded the Excellence Awards (Austria), which covered conference attendance and software purchase. All the aforementioned funds are unrelated to the project presented in this manuscript.
