# Supplementary material for: Targeting pyruvate kinase M2 (PKM2) reduces T cell pathogenicity in multiple sclerosis
Source: eBioMedicine. 2026 May 28;128:106314. doi: 10.1016/j.ebiom.2026.106314 (PMC13235379; doi:10.1016/j.ebiom.2026.106314)
Supplement: Supplementary Tables and Figures [file mmc1.docx]

**Targeting pyruvate kinase M2 (PKM2) reduces T cell pathogenicity in multiple sclerosis**

**TABLE OF CONTENTS**

SUPPLEMENTARY FIGURES and FIGURE LEGENDS_________________________________2

Supplementary Figure S1_____________________________________________________2

Supplementary Figure S2_____________________________________________________3

Supplementary Figure S3_____________________________________________________4

Supplementary Figure S4_____________________________________________________5

Supplementary Figure S5_____________________________________________________7

Supplementary Figure S6_____________________________________________________9

Supplementary Figure S7____________________________________________________10

Supplementary Figure S8____________________________________________________11

Supplementary Figure S9____________________________________________________12

SUPPLEMENTARY TABLES and TABLE LEGENDS _________________________________13

Supplementary Table S1 ____________________________________________________13

Supplementary Table S2 ____________________________________________________13

Supplementary Table S3 ____________________________________________________14

Supplementary Table S4 ____________________________________________________14

Supplementary Table S5 ____________________________________________________15

Supplementary Table S6 ____________________________________________________15

Supplementary Table S7 ____________________________________________________16

**SUPPLEMENTARY FIGURES and FIGURE LEGENDS**

**
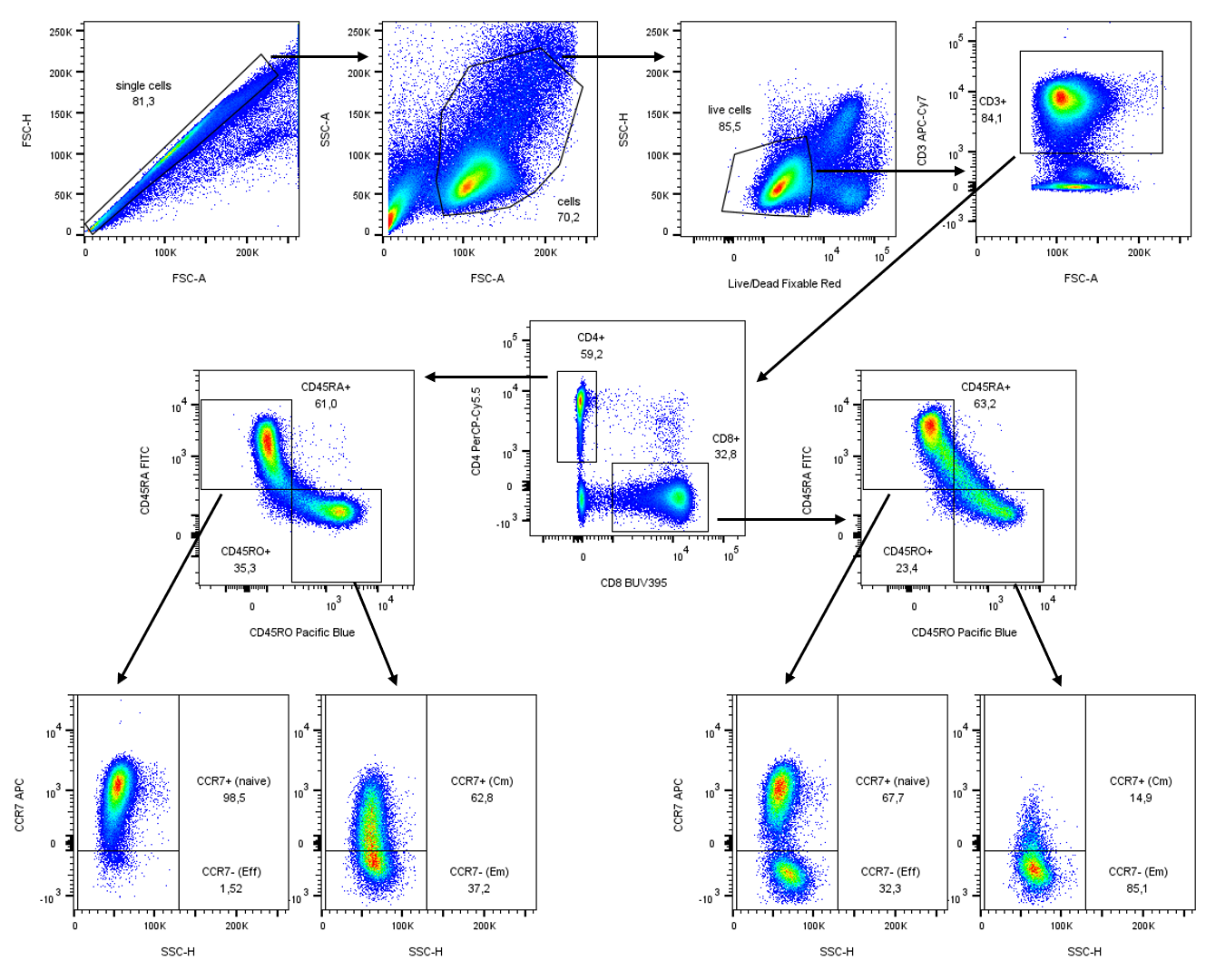
**

**Supplementary Figure S1. Representative flow cytometry plots for study cohort 1.** The gating strategy used to analyse PKM2 expression in circulating naïve, Cm, Em, and Eff T cells of one HC donor is shown.

**
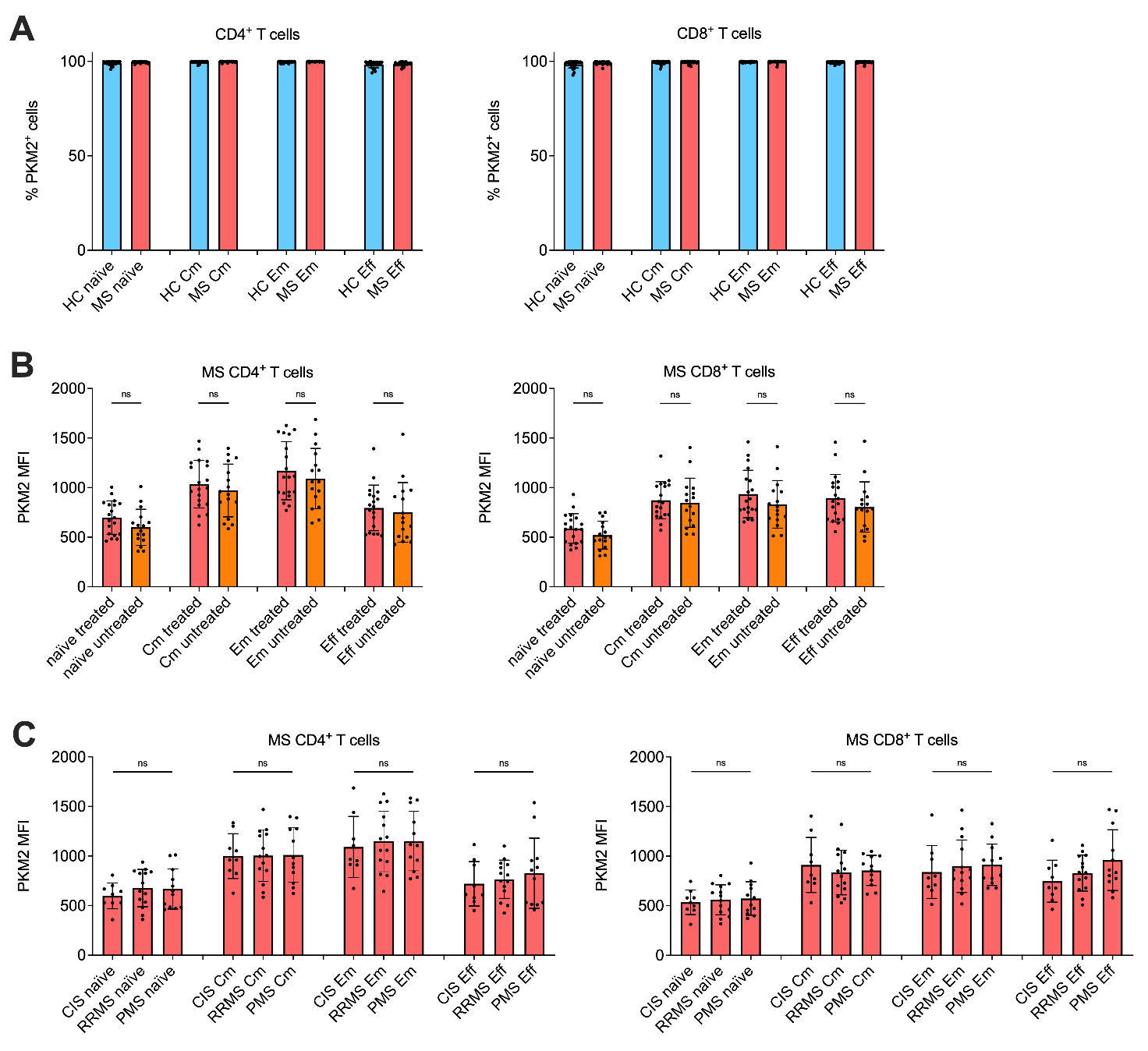
**

**Supplementary Figure S2. PKM2 expression in T cells from HCs and patients with MS.** (**A**) Percentage of PKM2^+^ naïve, Cm, Em and Eff T cells from HCs and individuals with MS. N=26 (HCs) and N=35 (MS). (**B**) PKM2 expression in T cells from untreated (naïve; N=16) and previously treated (N=19) patients with MS. All *p*-values were calculated by unpaired *t*-test, apart from the comparison between treated and untreated CD4^+^ Em cells, which was calculated by Mann-Whitney test. (**C**) PKM2 expression in T cell subsets of patients with CIS (N=9), RRMS (N=14) and PMS (N=12). All *p*-values were calculated by one-way Anova test with Tukey’s test for multiple comparisons. Data in (**A**-**C**) are presented as mean ± SD. ns, non-significant.


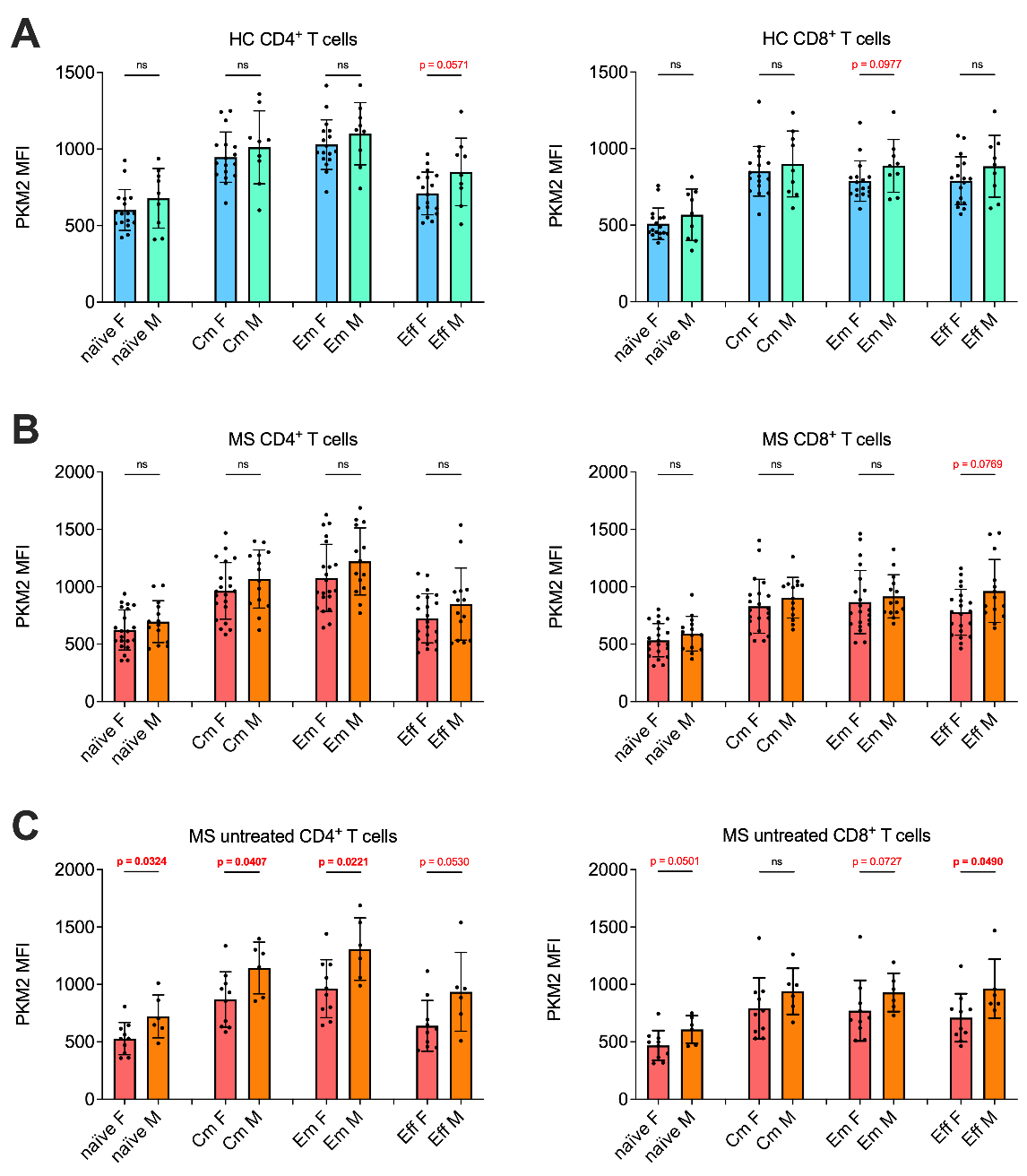


**Supplementary Figure S3. PKM2 expression in T cells from female *vs* male individuals.** (**A**) PKM2 expression in T cells from female (F; N=17) and male (M; N=9) HCs. All *p*-values were calculated by unpaired *t*-test, apart from the comparisons between female and male CD8^+^ naïve and Em cells, which was calculated by Mann-Whitney test. (**B**) PKM2 expression in T cells from female (N=21) and male (N=14) individuals with MS. All *p*-values were calculated by unpaired *t*-test, apart from the comparisons between female and male CD8^+^ Em and Eff cells, which was calculated by Mann-Whitney test. (**C**) PKM2 expression in T cells from female (N=10) and male (N=6) untreated (naïve) patients with MS. All *p*-values were calculated by unpaired *t*-test, apart from the comparison between female and male CD8^+^ Em cells, which was calculated by Mann-Whitney test. Data in (**A**-**C**) are presented as mean ± SD. *p*-values <0.05 are displayed in red, bold, while *p*-values <0.1 are displayed in red. ns, non-significant.


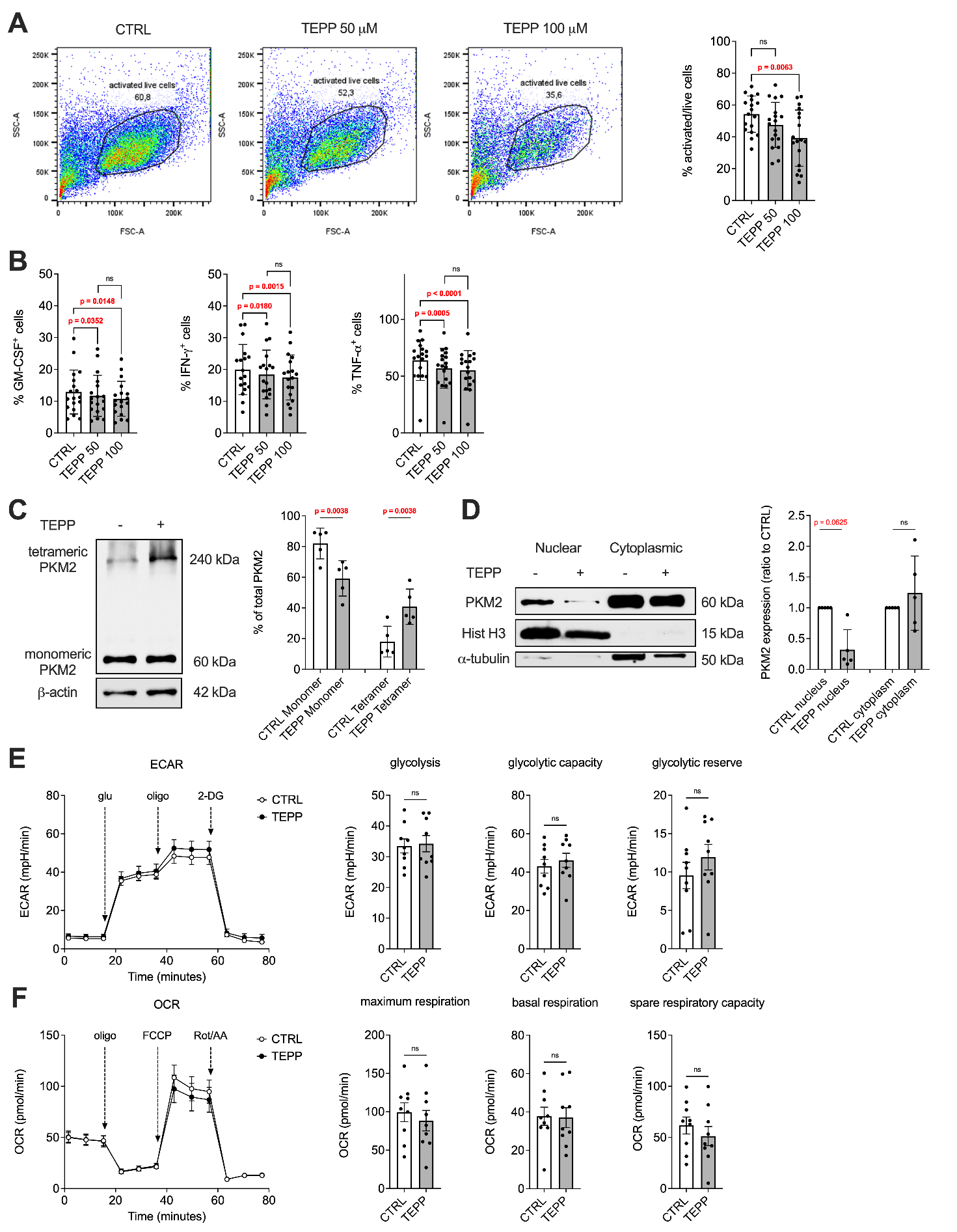


**Supplementary Figure S4. Effect of TEPP-46 on cultured PBMCs.** (**A**) PBMCs from HCs and patients with MS were activated for 5 days with anti-CD3/anti-CD28 antibodies in the presence of vehicle (CTRL) or TEPP-46 50 µM (TEPP 50) or 100 µM (TEPP 100). 5 days post-activation, cells were collected and activated live cells were gated based on their morphology. Left: representative plots showing PBMCs activated in the presence of vehicle or TEPP-46. Right: quantification of the percentage of activated live cells in the three conditions (N=18). *p*-values were calculated by one-way Anova with Dunnett’s test for multiple comparisons. (**B**) PBMCs from HCs and patients with MS were activated for 24 hours with anti-CD3/anti-CD28 antibodies in the presence of vehicle (CTRL) or TEPP-46 50 µM (TEPP 50) or 100 µM (TEPP 100). Cells were collected, restimulated with PMA/iono/BFA, and cytokine production was analysed by flow cytometry upon intracellular cytokine staining. N=18. *p*-values were calculated by one-way Anova with Tukey’s test for multiple comparisons (GM-CSF and IFN-γ) or by Friedman test with Dunn’s test for multiple comparisons (TNF-α). (**C**-**D**) HC PBMCs were activated for 3 days with anti-CD3/anti-CD28 antibodies in the presence of vehicle (-; CTRL) or TEPP-46 50 µM (+; TEPP). (**C**) Cells were collected, protein cross-linked, and PKM2 isomerisation was analysed by western blot. Left: representative western blot image. Right: Quantification of results (N=5). *p*-values were calculated by paired *t*-test for both monomer and tetramer comparisons. (**D**) Cells were collected, fractionated, and PKM2 cellular localisation was analysed by western blot. Left: representative image of PKM2 nuclear and cytoplasmic expression. Right: Quantification of results (N=5). *p*-values were calculated by Wilcoxon test for both nuclear and cytoplasmic comparisons. (**E**-**F**) HC PBMCs were activated for 3 days with anti-CD3/anti-CD28 antibodies in the presence of vehicle (CTRL) or TEPP-46 50 µM (TEPP). Cells were then collected and their glycolytic (**E**) and oxidative (**F**) metabolism was analysed via Seahorse assay. N=9. *p*-values were calculated by paired *t*-test for all comparisons. Data in (**A**-**D**) are presented as mean ± SD, while data in (**E**-**F**) are shown as mean ± standard error of the mean (SEM). *p*-values <0.05 are displayed in red, bold, while *p*-values <0.1 are displayed in red. ns, non-significant.

**
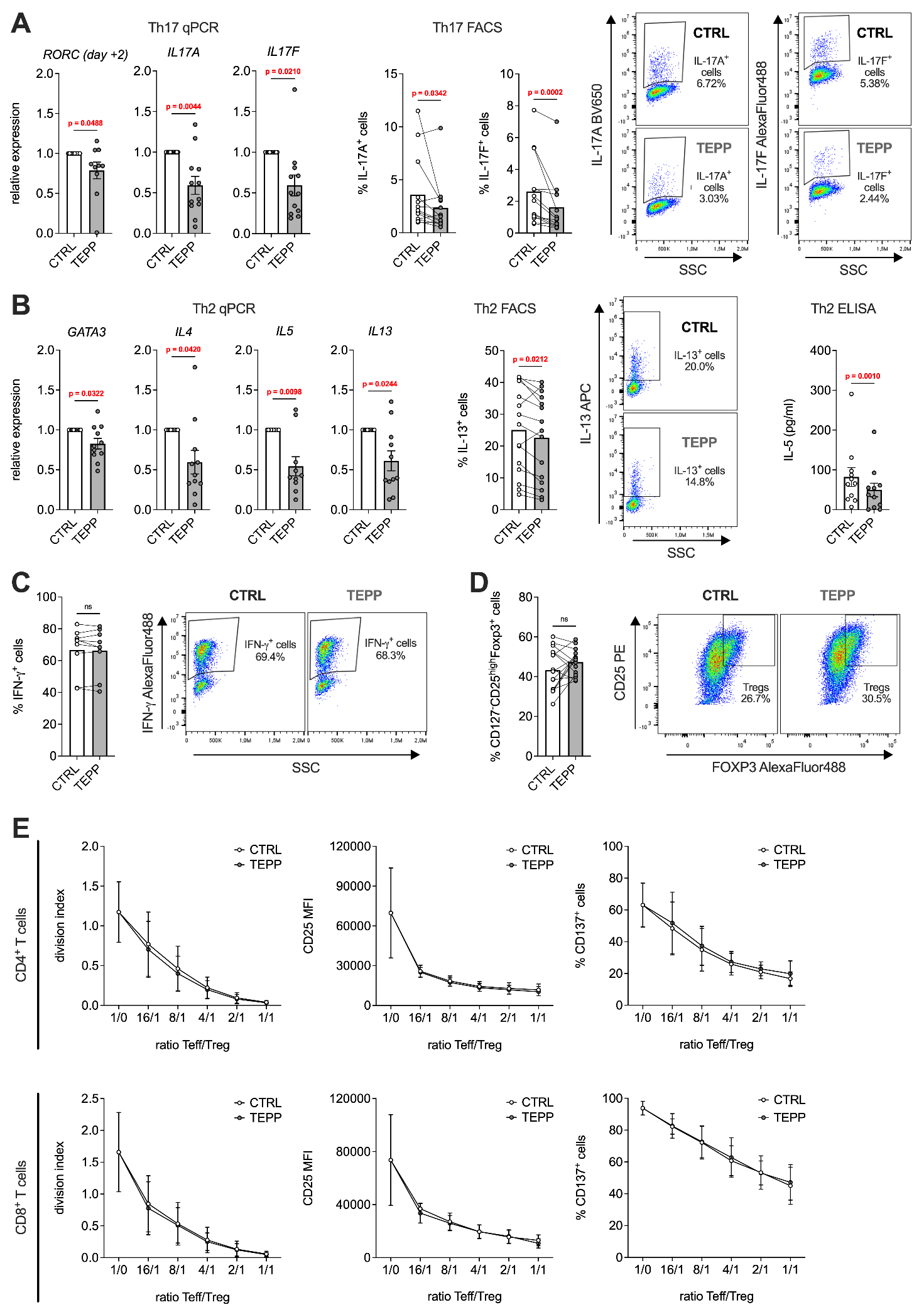
**

**Supplementary Figure S5. Effect of TEPP-46 on human CD4^+^ T cell polarisation and functionality.** (**A**-**D**) Human naïve CD4^+^ T cells were purified from PBMCs of healthy donors. Cells were polarised *in vitro* to Th1, Th17, Th2, and Tregs in the presence of vehicle (CTRL) or TEPP-46 50 µM (TEPP). (**A**) Analysis of Th17 cell polarisation by qPCR and flow cytometry (FACS). All *p*-values were calculated by Wilcoxon test. qPCR: N=10 (*RORC*) and N=12 (*IL17A* and *IL17F*). FACS: N=13. For analysis of *RORC* mRNA by qPCR, cells were collected at day +2 post-activation, to assess early reduction of *RORC* expression. (**B**) Analysis of Th2 cell polarisation by qPCR, FACS, and ELISA. All *p*-values were calculated by Wilcoxon test, apart from IL-13 FACS that was calculated by paired *t*-test. qPCR: N=10 (*IL5*) and N=11 (*GATA3*, *IL4*, *IL13*). FACS: N=15. ELISA: N=11. (**C**) Analysis of Th1 cell polarisation by flow cytometry. *p*-value was calculated by Wilcoxon test. N=9. (**D**) Analysis of Treg cell polarisation by flow cytometry. *p*-value was calculated by paired *t*-test. N=15. In the representative FACS plots, cells were pre-gated on CD127, and CD127^-^ cells are shown. In (**A**-**D**), qPCR and ELISA data are shown as mean ± SEM, while flow cytometry data are shown as mean ± SD. ns, non-significant. (**E**) Tregs induced in the presence of vehicle (CTRL) or TEPP-46 50 µM (TEPP) were mixed with CTV-stained CD3^+^ T cells purified from PBMCs of healthy donors. The mix was then activated with anti-CD3/anti-CD28 antibodies. After 5 days, the co-culture was collected and the following parameters were evaluated in CTV^+^ cells by flow cytometry: cell proliferation (shown as division index), expression of CD25 (shown as MFI of the staining) and expression of CD137 (shown as percentage of CD137^+^ cells), in both CTV^+^CD4^+^ and CTV^+^CD8^+^ T cells. Data are shown as mean ± SD and are from N=5 independent experiments.

**
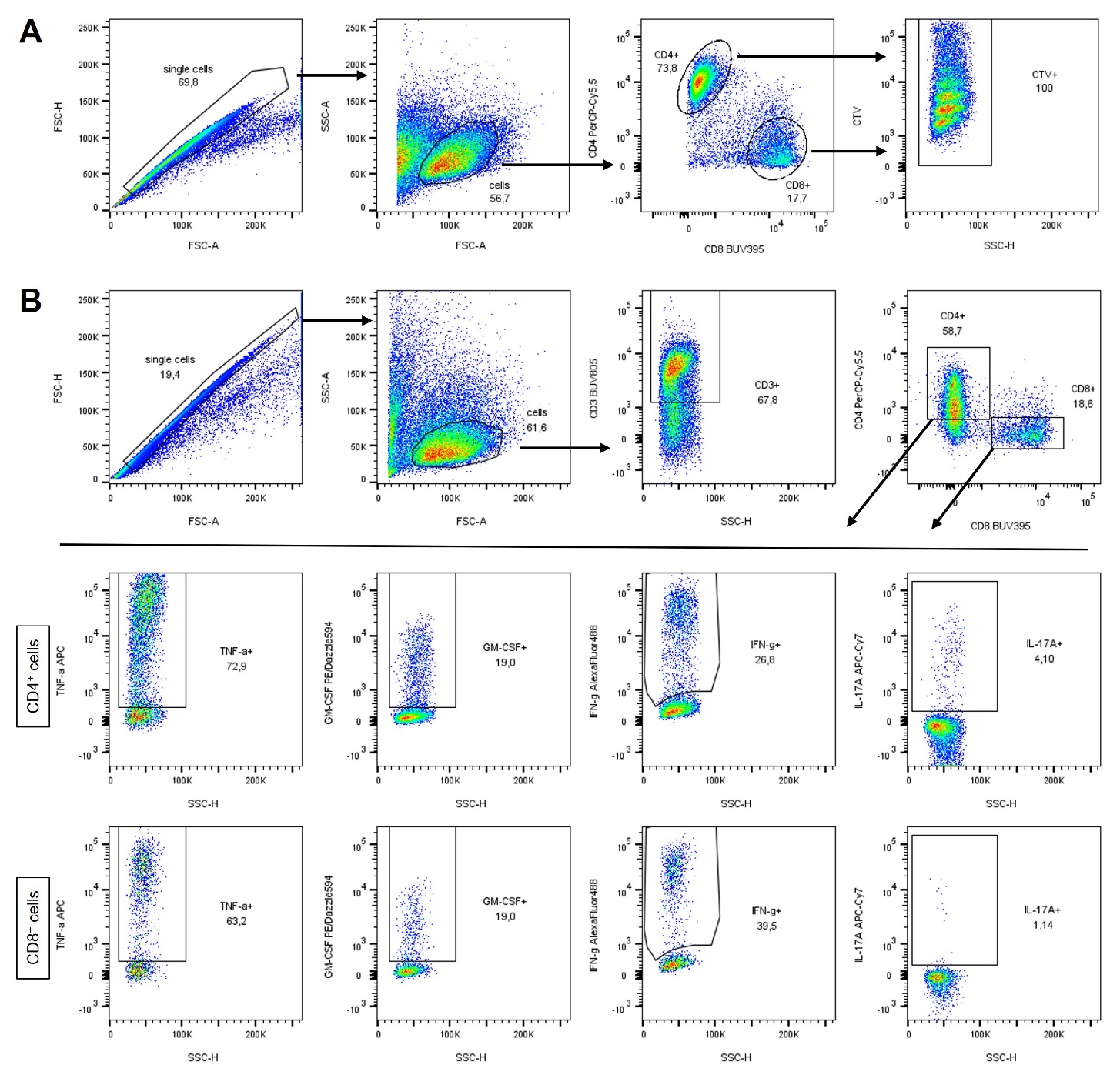
**

**Supplementary Figure S6. Representative flow cytometry plots for proliferation assays and for quantification of cytokine-producing cells.** (**A**) Representative gating strategy for the analysis of T cell proliferation in one patient with MS. (**B**) Representative gating strategy and cytokine plots from one patient with MS.


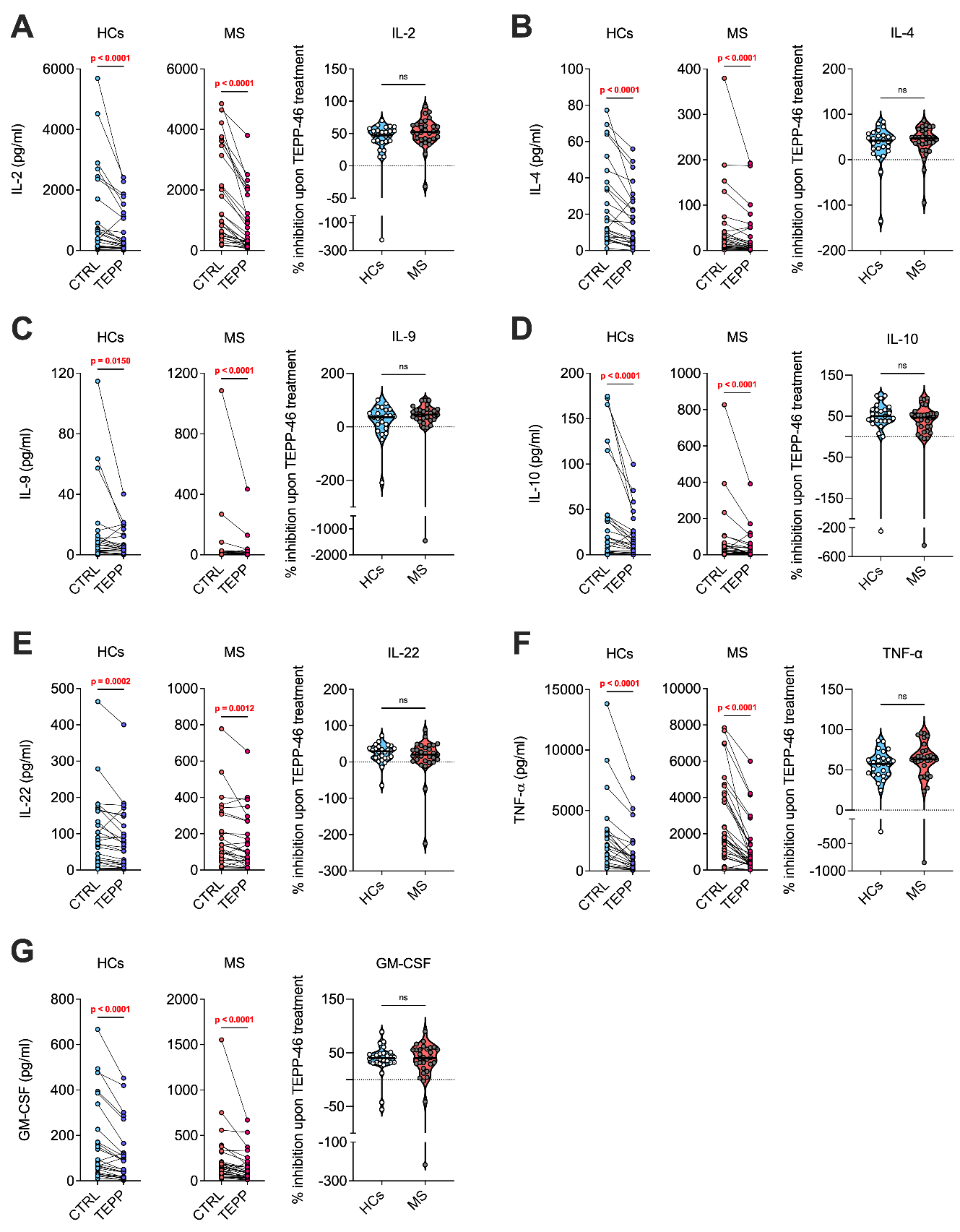


**Supplementary Figure S7. Cytokine quantification in supernatants from *in vitro* experiments.** PBMCs from HCs and patients with MS were activated for 24 hours with anti-CD3/anti-CD28 antibodies in the presence of vehicle (CTRL) or TEPP-46 50 µM (TEPP). Supernatants were collected and cytokine concentration was analysed by multiplex assay. In each panel, the left graphs show the cytokine concentrations in HC and MS sample supernatant, while the right graph shows the percentage of inhibition upon TEPP-46 treatment. The black horizontal line on top of the violin plots represents the median value. Results for IL-2 (**A**), IL-4 (**B**), IL-9 (**C**), IL-10 (**D**), IL-22 (**E**), TNF-α (**F**) and GM-CSF (**G**) are shown. In all panels, *p*-values were calculated by Wilcoxon test (left graphs) or by Mann-Whitney test (right graph). Data in (**A**-**G**) are from 26 HCs and 30 patients with MS. Samples from one patient with RRMS, one patient with CIS, and three patients with PMS could not be quantified due to technical issues during sample processing. ns, non-significant.


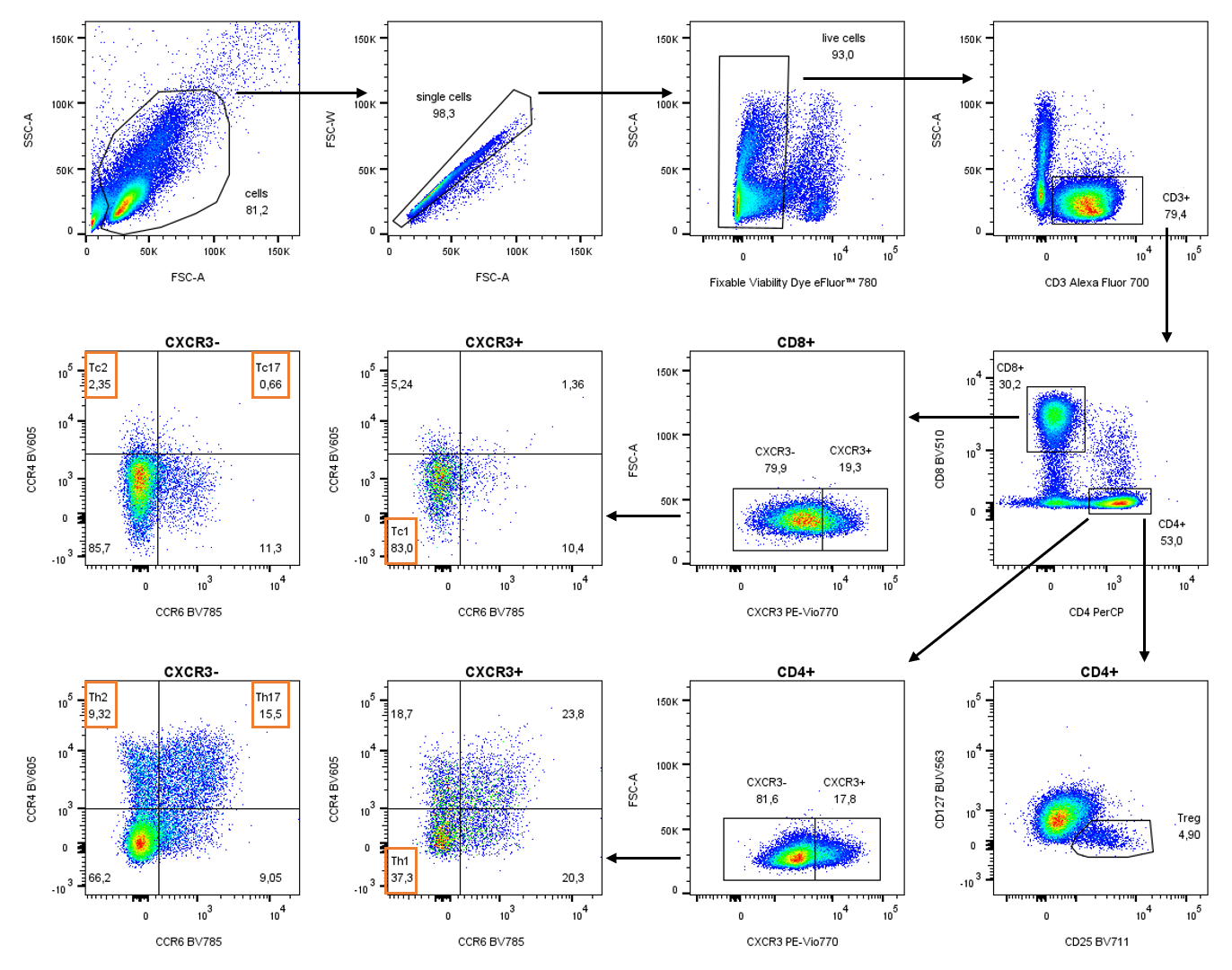


**Supplementary Figure S8. Representative flow cytometry plots for study cohort 2.** The gating strategy used to identify circulating Th1/Tc1, Th2/Tc2, Th17/Tc17 and Treg cells in one HC donor is shown.


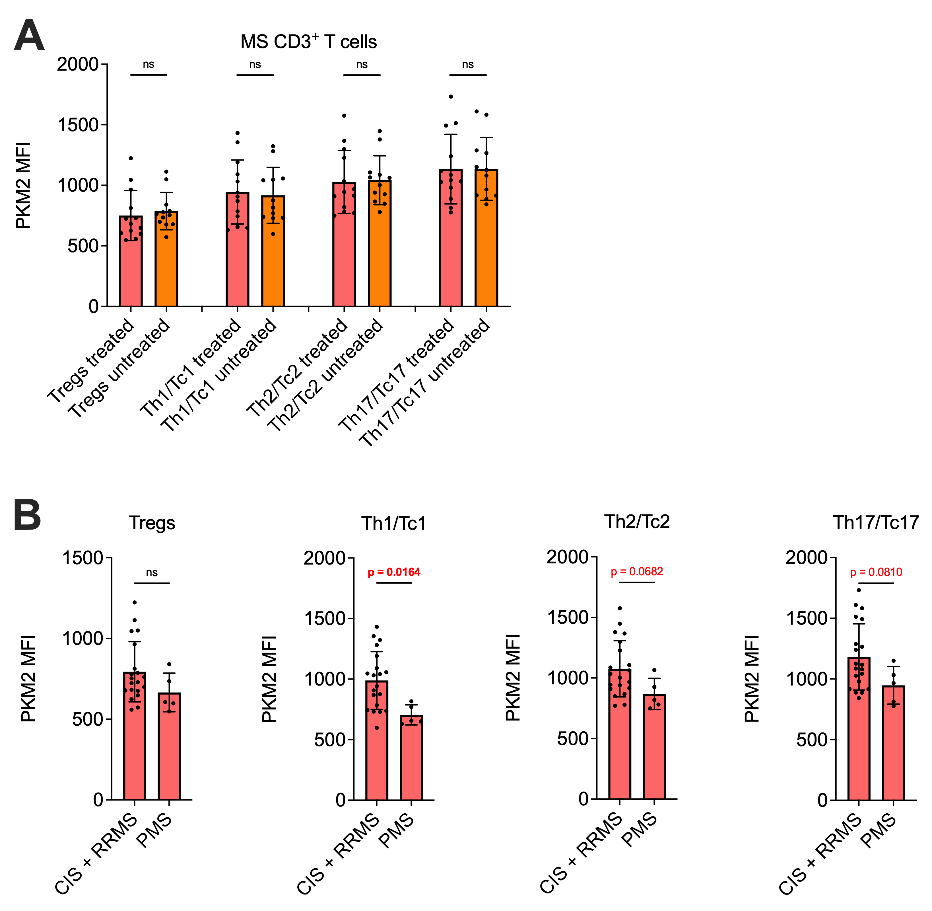


**Supplementary Figure S9. PKM2 expression in effector/memory (E/M) T cells from patients with MS.** (**A**) PKM2 expression in CD3^+^ T cells from untreated (naïve; N=12) and previously treated (N=13) patients with MS. All *p*-values were calculated by unpaired *t*-test, apart from the comparison between treated and untreated Tregs, which was calculated by Mann-Whitney test. (**B**) PKM2 expression in CD4^+^ (Tregs) or CD3^+^ E/M T cell subsets of individuals with CIS + RRMS (N=20) *vs* PMS (N=5). All *p*-values were calculated by unpaired *t*-test, apart from the comparison between CIS + RRMS and PMS Tregs, which was calculated by Mann-Whitney test. Data in (**A**-**B**) are presented as mean ± SD. *p*-values <0.05 are displayed in red, bold, while *p*-values <0.1 are displayed in red. ns, non-significant.

**SUPPLEMENTARY TABLES and TABLE LEGENDS**

| **Subset** | **beta** | **95% CI** | ***p*-value** |
| --- | --- | --- | --- |
| CD4 naïve | 16.01 | -71.2 - 103.22 | 0.71 |
| CD4 Cm | 26.38 | -91.11 - 143.88 | 0.66 |
| CD4 Em | 68.04 | -61.17 - 197.26 | 0.30 |
| CD4 Eff | -3.11 | -116.13 - 109.92 | 0.96 |
| CD8 naïve | 18.85 | -52.18 - 89.87 | 0.60 |
| CD8 Cm | -14.95 | -119.34 - 89.43 | 0.78 |
| CD8 Em | 51.45 | -54.08 - 156.99 | 0.33 |
| CD8 Eff | 12.34 | -94.3 - 118.98 | 0.82 |

**Supplementary Table S1. Comparison between PKM2 expression in T cell subsets of HCs *vs* patients with MS, adjusted for sex and age (cohort 1).** This Table refers to Figure 1E and 1F of the manuscript. beta: regression coefficient for the comparison of CD4/CD8 values between HCs and patients with MS, adjusted for sex and age. 95% CI: 95% confidence interval of beta.

|  | **Age at baseline**  **HCs (N=26)** | | **Age at baseline**  **Patients with MS (N=35)** | |
| --- | --- | --- | --- | --- |
| **Subset** | **r** | ***p*-value** | **r** | ***p*-value** |
| CD4 naïve | 0.05 | 0.82 | 0.24 | 0.17 |
| CD4 Cm | 0.07 | 0.73 | 0.15 | 0.40 |
| CD4 Em | 0.10 | 0.65 | 0.17 | 0.33 |
| CD4 Eff | 0.03 | 0.87 | 0.31* | 0.07 |
| CD8 naïve | 0.23* | 0.27 | 0.22 | 0.21 |
| CD8 Cm | 0.11 | 0.61 | 0.10 | 0.58 |
| CD8 Em | -0.01* | 0.97 | 0.34* | 0.05 |
| CD8 Eff | -0.06 | 0.78 | 0.38 | **0.03** |

**Supplementary Table S2. Correlation analysis between PKM2 expression and age in HCs and patients with MS, adjusted for sex using partial correlation.** This Table refers to Table 2 of the manuscript. r: Pearson partial correlation coefficient adjusted for sex, except for *Spearman partial correlation coefficient adjusted for sex.

|  | **Disease duration**  **(months; N=35)** | | **EDSS (N=33)** | |
| --- | --- | --- | --- | --- |
| **Subset** | **r** | ***p*-value** | **r** | ***p*-value** |
| CD4 naïve | -0.11 | 0.55 | -0.05 | 0.80 |
| CD4 Cm | -0.18 | 0.32 | -0.13 | 0.48 |
| CD4 Em | -0.04 | 0.82 | -0.03 | 0.88 |
| CD4 Eff | -0.28 | 0.11 | -0.14 | 0.44 |
| CD8 naïve | -0.14 | 0.44 | -0.05 | 0.81 |
| CD8 Cm | -0.16 | 0.37 | -0.04 | 0.83 |
| CD8 Em | -0.03 | 0.87 | -0.02 | 0.90 |
| CD8 Eff | -0.06 | 0.76 | 0.0001 | 1.00 |

**Supplementary Table S3. Correlation analysis between PKM2 expression and disease activity in patients with MS, adjusted for sex and age using partial correlation.** This Table refers to Table 3 of the manuscript. r: Spearman partial correlation coefficient adjusted for sex and age.

|  | **Disease duration (months)** | | **Annualised relapse rate** | | **Time from last relapse to**  **baseline (months)** | |
| --- | --- | --- | --- | --- | --- | --- |
| **Subset** | **r^$^** | ***p*-value** | **r*** | ***p*-value** | **r^$^** | ***p*-value** |
| CD4 naïve | 0.41 | 0.18 | 0.31 | 0.33 | 0.06 | 0.86 |
| CD4 Cm | 0.56 | 0.06 | 0.22 | 0.49 | 0.00 | 0.99 |
| CD4 Em | 0.66 | **0.02** | 0.27 | 0.40 | 0.14 | 0.67 |
| CD4 Eff | 0.48 | 0.12 | 0.34 | 0.28 | 0.03 | 0.93 |
| CD8 naïve | 0.50 | 0.10 | 0.23 | 0.47 | 0.05 | 0.88 |
| CD8 Cm | 0.18 | 0.59 | 0.09 | 0.78 | -0.17 | 0.61 |
| CD8 Em | 0.62 | **0.03** | 0.28 | 0.39 | 0.36 | 0.25 |
| CD8 Eff | 0.47 | 0.12 | 0.29 | 0.36 | 0.11 | 0.74 |

**Supplementary Table S4. Correlation analysis between PKM2 expression and disease course in patients with RRMS (N=14), adjusted for sex and age using partial correlation.** This Table refers to Table 4 of the manuscript. r^$^: Pearson partial correlation coefficient adjusted for age and sex. r*: Spearman partial correlation coefficient adjusted for sex and age.

| **Cytokine** | **HCs** | **Patients with MS** | ***p*-value** |
| --- | --- | --- | --- |
| IL-2 | 479.46 [145.00 – 1868.15] | 955.11 [467.78 – 3221.18] | **0.030** |
| IL-4 | 14.14 [7.22 – 39.27] | 19.60 [7.76 – 41.15] | 0.702 |
| IL-5 | 6.71 [2.08 – 43.51] | 19.69 [5.31 – 48.24] | 0.265 |
| IL-9 | 6.39 [1.75 – 11.09] | 8.86 [2.04 – 19.29] | 0.367 |
| IL-10 | 18.93 [3.63 – 43.08] | 20.59 [5.14 – 55.98] | 0.509 |
| IL-13 | 24.68 [12.87 – 82.29] | 28.43 [7.79 – 71.45] | 0.678 |
| IL-17A | 4.52 [1.23 – 15.59] | 9.30 [2.98 – 23.47] | 0.088 |
| IL-17F | 2.04 [0.70 – 5.64] | 2.72 [0.98 – 5.78] | 0.268 |
| IL-22 | 77.32 [27.31 – 163.61] | 116.71 [55.22 – 236.70] | 0.113 |
| IFN-γ | 2621.57 [987.57 – 11219.52] | 4983.50 [1390.49 – 11655.95] | 0.488 |
| TNF-α | 1834.14 [594.00 – 3239.22] | 1814.22 [1034.57 – 4359.33] | 0.468 |
| GM-CSF | 79.36 [32.73 – 254.66] | 152.94 [69.78 – 236.66] | 0.156 |

**Supplementary Table S5. Cytokine levels in supernatants from *in vitro* experiments.** Data are reported as cytokine concentration in pg/ml (median and [interquartile range]) and are from N=26 (HCs) or N=30 (patients with MS). *p*-values were calculated by Mann-Whitney test.

| **CD4^+^ T cells** | | | |
| --- | --- | --- | --- |
| **Subset** | **beta** | **95% CI** | ***p*-value** |
| Tregs | 27.73 | -68.97 - 124.42 | 0.57 |
| Th1 | 25.85 | -91.25 - 142.95 | 0.66 |
| Th2 | 23.60 | -102.15 - 149.34 | 0.71 |
| Th17 | 70.70 | -70.68 - 212.08 | 0.32 |
| **CD8^+^ T cells** | | | |
| **Subset** | **beta** | **95% CI** | ***p*-value** |
| Tc1 | 6.14 | -123.07 - 135.36 | 0.92 |
| Tc2 | 39.38 | -99.8 - 178.56 | 0.57 |
| Tc17 | 96.96 | -67.79 - 261.71 | 0.24 |
| **CD3^+^ T cells** | | | |
| **Subset** | **beta** | **95% CI** | ***p*-value** |
| Th1/Tc1 | 14.03 | -122.22 - 150.29 | 0.84 |
| Th2/Tc2 | 8.80 | -116.63 - 134.23 | 0.89 |
| Th17/Tc17 | 52.87 | -97.07 - 202.81 | 0.48 |

**Supplementary Table S6. Comparison between PKM2 expression in T cell subsets of HCs vs patients with MS, adjusted for sex and age (cohort 2).** This Table refers to Figure 6D of the manuscript. beta: regression coefficient for the comparison of CD4/CD8 values between HCs and patients with MS, adjusted for sex and age. 95% CI: 95% confidence interval of beta.

| **REAGENT or RESOURCE** | **SOURCE** | **IDENTIFIER** |
| --- | --- | --- |
| **Antibodies** | | |
| APC/Cyanine7 anti-human CD3 Antibody (clone SK7) | BioLegend | Cat# 344818;  RRID:AB_10645474 |
| PerCP/Cyanine5.5 anti-human CD4 Antibody (clone SK3) | BioLegend | Cat# 344608;  RRID:AB_1953236 |
| BD Horizon™ BUV395 Mouse Anti-Human CD8 (clone RPA-T8) | BD Biosciences | Cat# 563795;  RRID:AB_2722501 |
| FITC anti-human CD45RA (clone HI100) | BioLegend | Cat# 983002;  RRID:AB_2650650 |
| Pacific Blue™ anti-human CD45RO Antibody (clone UCHL1) | BioLegend | Cat# 304216;  RRID:AB_493659 |
| APC anti-human CD197 (CCR7) Antibody (clone G043H7) | BioLegend | Cat# 353214;  RRID:AB_10917387 |
| PKM2 (D78A4) XP® Rabbit mAb (PE Conjugate) | Cell Signaling Technology | Cat# 89367S;  RRID:AB_2800137 |
| Rabbit (DA1E) mAb IgG XP® Isotype Control (PE Conjugate) | Cell Signaling Technology | Cat# 5742S;  RRID:AB_10694219 |
| Alexa Fluor® 700 anti-human CD3 Antibody (clone SK7) | BioLegend | Cat# 344822;  RRID:AB_2563420 |
| PerCP anti-human CD4 Antibody (clone SK3) | BioLegend | Cat# 344624;  RRID:AB_2563326 |
| Brilliant Violet 510™ anti-human CD8a Antibody (clone HIT8a) | BioLegend | Cat# 300933;  RRID:AB_2814114 |
| CD183 (CXCR3) Antibody, anti-human, PE-Vio® 770, REAfinity™ (clone REA232\|G025H7) | Miltenyi Biotec | Cat# 130-120-453;  RRID:AB_2784024 |
| Brilliant Violet 605™ anti-human CD194 (CCR4) Antibody (clone L291H4) | BioLegend | Cat# 359417;  RRID:AB_2562482 |
| Brilliant Violet 785™ anti-human CD196 (CCR6) Antibody (clone G034E3) | BioLegend | Cat# 353421;  RRID:AB_2561372 |
| CD127 Monoclonal Antibody (eBioRDR5), Brilliant Ultra Violet™ 563, eBioscience™ | Thermo Fisher Scientific | Cat# 365-1278-42;  RRID:AB_2920973 |
| Brilliant Violet 711™ anti-human CD25 Antibody (clone M-A251) | BioLegend | Cat# 356137;  RRID:AB_2632780 |
| BD Horizon™ BUV805 Mouse Anti-Human CD3 (clone SK7) | BD Biosciences | Cat# 612893;  RRID:AB_2870181 |
| Alexa Fluor® 488 anti-human IFN-γ Antibody (clone 4S.B3) | BioLegend | Cat# 502515;  RRID:AB_493029 |
| PE/Dazzle™ 594 anti-mouse GM-CSF Antibody (clone MP1-22E9) | BioLegend | Cat# 505422;  RRID:AB_2814425 |
| Brilliant Violet 421™ anti-human IL-17A Antibody (clone BL168) | BioLegend | Cat# 512322;  RRID:AB_11218604 |
| APC anti-human TNF-α Antibody (clone MAb11) | BioLegend | Cat# 502912;  RRID:AB_315264 |
| PE anti-human CD25 Antibody (clone BC96) | BioLegend | Cat# 302606;  RRID:AB_314276 |
| Alexa Fluor® 647 anti-human CD127 (IL-7Rα) Antibody (clone A019D5) | BioLegend | Cat# 351318;  RRID:AB_10896063 |
| Alexa Fluor® 488 anti-mouse/rat/human FOXP3 Antibody (clone 150D) | BioLegend | Cat# 320012;  RRID:AB_439748 |
| Brilliant Violet 605™ anti-human CD137 (4-1BB) Antibody (clone 4B4-1) | BioLegend | Cat# 309822;  RRID:AB_2565997 |
| BD Horizon™ BV421 Rat Anti-Human IL-4 (clone MP4-25D2) | BD Biosciences | Cat# 564110;  RRID:AB_2738599 |
| APC anti-human IL-13 Antibody (clone JES10-5A2) | BioLegend | Cat# 501908;  RRID:AB_315203 |
| Alexa Fluor® 488 anti-human IL-17F Antibody (clone Poly5166) | BioLegend | Cat# 516604;  RRID:AB_10720816 |
| Brilliant Violet 650™ anti-human IL-17A Antibody (clone BL168) | BioLegend | Cat# 512346;  RRID:AB_3683467 |
| APC/Cyanine7 anti-human IL-17A Antibody (clone BL168) | BioLegend | Cat# 512320;  RRID:AB_10613103 |
| beta-Actin (13E5) Rabbit Monoclonal Antibody | Cell Signaling Technology | Cat# 4970;  RRID:AB_2223172 |
| alpha-Tubulin Antibody | Cell Signaling Technology | Cat# 2144;  RRID:AB_2210548 |
| Histone H3 (D1H2) Rabbit Monoclonal Antibody | Cell Signaling Technology | Cat# 4499;  RRID:AB_10544537 |
| Anti-rabbit IgG, HRP-linked Antibody | Cell Signaling Technology | Cat# 7074;  RRID:AB_2099233 |
| PKM2 (D78A4) XP® Rabbit mAb | Cell Signaling Technology | Cat# 4053;  RRID:AB_1904096 |
| Ultra-LEAF™ Purified anti-human CD28 Antibody (clone CD28.2) | BioLegend | Cat# 302934;  RRID:AB_11148949 |
| Ultra-LEAF™ Purified anti-human CD3 Antibody (clone UCHT1) | BioLegend | Cat# 300438;  RRID:AB_11146991 |
| Purified anti-human IFN-γ Antibody (clone B27) | BioLegend | Cat# 506502;  RRID:AB_315435 |
| **Chemicals, peptides, and recombinant proteins** | | |
| DSS (disuccinimidyl suberate), No-Weigh™ Format | Thermo Fisher Scientific | Cat# A39267;  CAS: 68528-80-3 |
| 4x Laemmli Sample Buffer | Bio-Rad | Cat# 1610747 |
| Recombinant Human Interleukin 6 | ImmunoTools | Cat# 11340060 |
| Recombinant Human Interleukin-23 | ImmunoTools | Cat# 11340233 |
| Recombinant Human Transforming Growth Factor-beta 1 | ImmunoTools | Cat# 11343161 |
| Human IL-1 beta Recombinant Protein | PeproTech | Cat# 200-01B-2UG |
| Recombinant Human Interleukin-2 | ImmunoTools | Cat# 11340023 |
| Recombinant Human IL-12 Protein | R&D Systems | Cat# 219-IL-005/CF |
| Recombinant Human Interleukin-4 | ImmunoTools | Cat# 11340043 |
| D-(+)-Glucose solution | Sigma-Aldrich | Cat# G8644;  CAS: 50-99-7 |
| Sodium pyruvate solution | Sigma-Aldrich | Cat# S8636;  CAS: 113-24-6 |
| D(+)-Glucose CELLPURE® ≥98 %, anhydrous | Carl ROTH | Cat# HN06.3;  CAS: 50-99-7 |
| Oligomycin | Sigma-Aldrich | Cat# O4876;  CAS: 1404-19-9 |
| 2-Deoxy-D-glucose | Sigma-Aldrich | Cat# D8375;  CAS: 154-17-6 |
| Seahorse XF RPMI medium | Agilent | Cat# 103576-100 |
| Intracellular Staining Permeabilization Wash Buffer (10X) | BioLegend | Cat# 421002 |
| Poly-D-Lysine | Thermo Fisher Scientific | Cat# A3890401 |
| L-Glutamine | PAA Laboratories | Cat# M11-004 |
| eBioscience™ Foxp3 / Transcription Factor Staining Buffer Set | Thermo Fisher Scientific | Cat# 00-5523-00 |
| TEPP-46 | Sigma-Aldrich | Cat# 505487;  CAS:1221186-53-3 |
| Cell Activation Cocktail (without Brefeldin A) | BioLegend | Cat# 423302 |
| Brefeldin A Solution (1,000X) | BioLegend | Cat# 420601 |
| Fixation Buffer | BioLegend | Cat# 420801 |
| BD Horizon™ Brilliant Stain Buffer | BD Biosciences | Cat# 563794 |
| Phorbol 12-myristate 13-acetate | Sigma-Aldrich | Cat# P8139;  CAS: 16561-29-8 |
| Ionomycin calcium salt | Sigma-Aldrich | Cat# I0634;  CAS: 56092-82-1 |
| Brefeldin A | Sigma-Aldrich | Cat# B7651;  CAS: 20350-15-6 |
| Clarity Western ECL Substrate, 500 ml | Bio-Rad | Cat# 1705061 |
| X-VIVO 15^TM^ | Lonza | Cat# 02-060Q |
| Bovine Serum Albumin (BSA) | Carl ROTH | Cat# 8076.4;  CAS: 9048-46-8 |
| Powdered milk | Carl ROTH | Cat# T145.2;  CAS: 68514-61-4 |
| Substrate Reagent Pack | R&D Systems | Cat# DY999 |
| Stop Solution 2N Sulfuric Acid | R&D Systems | Cat# DY994 |
| **Commercial assays** | | |
| LIVE/DEAD™ Fixable Red Dead Cell Stain Kit | Thermo Fisher Scientific | Cat# L34971 |
| eBioscience™ Fixable Viability Dye eFluor™ 780 | Thermo Fisher Scientific | Cat# 65-0865-18 |
| CellTrace™ Violet Cell Proliferation Kit | Thermo Fisher Scientific | Cat# C34557 |
| PARIS™ Kit | Thermo Fisher Scientific | Cat# AM1921 |
| EasySep™ Human Naïve CD4+ T Cell Isolation Kit | STEMCELL Technologies | Cat# 17555 |
| Pan T Cell Isolation Kit, human | Miltenyi Biotec | Cat# 130-096-535 |
| High-Capacity cDNA Reverse Transcription Kit | Thermo Fisher Scientific | Cat# 4368813 |
| PowerUp™ SYBR™ Green Master Mix | Thermo Fisher Scientific | Cat# A25742 |
| PureLink™ RNA Mini Kit | Thermo Fisher Scientific | Cat# 12183025 |
| innuPREP RNA Mini Kit 2.0 | IST Innuscreen | Cat# 845-KS-2040250 |
| Trans-Blot Turbo RTA Mini 0.2 µm Nitrocellulose Transfer Kit | Bio-Rad | Cat# 1704270 |
| Seahorse XF Cell Mito Stress Test Kit | Agilent | Cat# 103015-100 |
| Seahorse XF Pro M FluxPak Mini | Agilent | Cat# 103777-100 |
| LEGENDplex™ HU Essential Immune Response Panel Detection Abs | BioLegend | Cat# 740931 |
| LEGENDplex™ HU Essential Immune Response Panel Standard | BioLegend | Cat# 740932 |
| LEGENDplex™ HU Th Cytokine Panel (12-plex) | BioLegend | Cat# 741028 |
| LEGENDplex™ Hu Anti-Virus Response Panel 1 Detection Abs V02 | BioLegend | Cat# 741273 |
| LEGENDplex™ Hu Anti-Virus Response Panel 1 Standard V02 | BioLegend | Cat# 741274 |
| LEGENDplex™ Human Free Active TGF-β1 Capture Bead B9, 13X | BioLegend | Cat# 740944 |
| LEGENDplex™ Human GM-CSF Capture Bead B5, 13X | BioLegend | Cat# 740362 |
| Human IL-5 DuoSet ELISA | R&D Systems | Cat# DY205-05 |
| **Oligonucleotides** | | |
| human *IL4* FWD: CAGCTGATCCGATTCCTGAAA human *IL4* REV: GTACTCTGGTTGGCTTCCTTC | Thermo Fisher Scientific | N/A |
| human *IL5* FWD: GAGACTCTGAGGATTCCTGTTC  human *IL5* REV: GACTCTCCAGTGTGCCTATTC | Thermo Fisher Scientific | N/A |
| human *IL13* FWD: GCTGAGCAACATCACACAAG human *IL13* REV: AATCCAGGGCTACACAGAAC | Thermo Fisher Scientific | N/A |
| human *IL17a* FWD: ACCGGAATACCAATACCAATCC human *IL17a* REV: GGATATCTCTCAGGGTCCTCAT | Thermo Fisher Scientific | N/A |
| human *IL17f* FWD: TGTCACGTAACATCGAGAGC human *IL17f* REV: CATTGATGCAGCCCAAGTTC | Thermo Fisher Scientific | N/A |
| human *TBP* FWD: CTTCGGAGAGTTCTGGGATTG human *TBP* REV: CACGAAGTGCAATGGTCTTTAG | Thermo Fisher Scientific | N/A |
| human *RORγt* FWD: ATGGAAGTGGTGCTGGTTAG human *RORγt* REV: GGAGTGGGAGAAGTCAAAGATG | Thermo Fisher Scientific | N/A |
| human *GATA3* FWD: GAACTGTCAGACCACCACAA human *GATA3* REV: GCCTTCCTTCTTCATAGTCAGG | Thermo Fisher Scientific | N/A |
| human *Tbet (Tbx21)* FWD: CAACAACCCCTTTGCCAAAG human *Tbet (Tbx21)* REV: TCCCCCAAGCAGTTGACAGT | Thermo Fisher Scientific | N/A |
| human IFNγ FWD: CTCTTCTTGGATATCTGGAGGAAC human IFNγ REV: GACTTCAAAGAGTCTGAGGTAGAA | Thermo Fisher Scientific | N/A |
| **Software and algorithms** | | |
| R software (version 3.6.2) | N/A | <https://www.r-project.org/> |
| Image Lab (version 6.1) | Bio-Rad | <https://www.bio-rad.com/de-at/product/image-lab-software?ID=KRE6P5E8Z> |
| FlowJo (version 10.10.0) | FlowJO | <https://www.flowjo.com/> |
| Prism (version 10.5.0) | GraphPad | <https://www.graphpad.com/scientific-software/prism/> |

**Supplementary Table S7. List of reagents used in the study.**
